# The double-edged nature of nicotine: toxicities and therapeutic potentials

**DOI:** 10.3389/fphar.2024.1427314

**Published:** 2024-08-14

**Authors:** Yun Cao, Jiali Sun, Xiaofeng Wang, Xiaoyu Zhang, Huijuan Tian, Lingling Huang, Ze Huang, Yaping Zhang, Jin Zhang, Lin Li, Shun Zhou

**Affiliations:** ^1^ Key Laboratory of Combustion & Pyrolysis Study of CNTC, China Tobacco Anhui Industrial Co., Ltd., Hefei, China; ^2^ The Institute of Flexible Electronics (IFE, Future Technologies), Xiamen University, Xiamen, China; ^3^ Department of Obstetrics, Women and Children’s Hospital, School of Medicine, Xiamen University, Xiamen, China; ^4^ Key Laboratory of Aerosol Analysis Regulation and Biological Effects of Anhui Province, China Tobacco Anhui Industrial Co., Ltd., Hefei, China

**Keywords:** nicotine, double-edged effect, toxicity, therapeutic potential, pharmacodynamics

## Abstract

Nicotine is the primary addictive component of cigarette smoke and is associated with various smoking-related diseases. However, recent research has revealed its broader cognitive-enhancing and anti-inflammatory properties, suggesting its potential therapeutic applications in several conditions. This review aims to examine the double-edged nature of nicotine, encompassing its positive and negative effects. We provide a concise overview of the physiochemical properties and pharmacology of nicotine, including insights into nicotine receptors. Therefore, the article is divided into two main sections: toxicity and therapeutic potential. We comprehensively explored nicotine-related diseases, focusing on specific signaling pathways and the underlying mechanisms that contribute to its effects. Furthermore, we addressed the current research challenges and future development perspectives. This review aims to inspire future researchers to explore the full medical potential of nicotine, which holds significant promise for the clinical management of specific diseases.

## Graphical Abstract

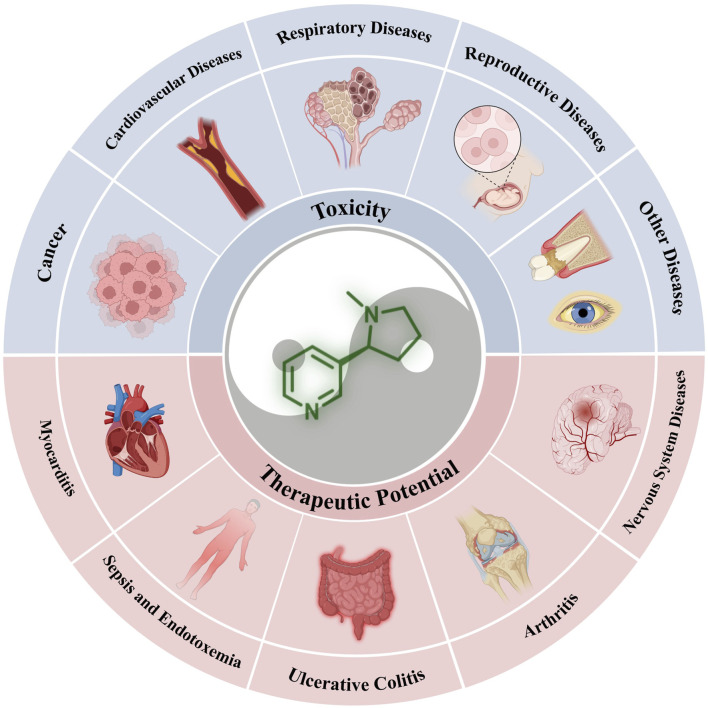

## 1 Introduction

Nicotine, an alkaloid naturally occurring in plants of the nightshade family, is a controversial molecule within the tobacco research community (Xu and Deng, 2004). However, it has notably garnered recognition as a therapeutic agent with the potential to treat various diseases. Nicotine has a double-edged nature and significantly affects the occurrence and progression of various diseases when introduced into the human body. For instance, nicotine binds to nicotinic acetylcholine receptors (nAChRs) in cancer development. It activates multiple downstream pathways, contributing to the regulation of cancer cell proliferation, phenotypic transformation, and cell migration, thereby damaging the human body (Xu and Deng, 2004). However, nicotine can interact with nAChR on immune cells in diseases such as sepsis and endotoxemia, reducing the damage caused by inflammatory cytokines and excessive immune responses, thus playing a beneficial role (Jull et al., 2001; van Westerloo et al., 2005). Therefore, it becomes crucial to differentiate the effects of nicotine from those of other toxic substances in cigarettes to fully explore its medicinal potential. This article objectively assesses the toxicity and therapeutic value of nicotine and offers a valuable reference for researchers.

This review focuses on the dual nature of nicotine, examining both its positive and negative characteristics. First, we offer a concise introduction to the physicochemical properties of nicotine and its crucial receptors on cell membranes. Subsequently, its toxic effects and therapeutic potential have been discussed. We categorized the diseases associated with nicotine toxicity into five groups: cancers, cardiovascular diseases, respiratory system diseases, reproductive system diseases, and other diseases. In the therapeutic section, we categorized the relevant diseases as nervous and immune system diseases, considering the wide-ranging cognitive-enhancing and anti-inflammatory properties of nicotine. Finally, we reflect on the current challenges and discuss potential future research directions.

## 2 Nicotine and its physiochemical properties

**FIGURE 1:**
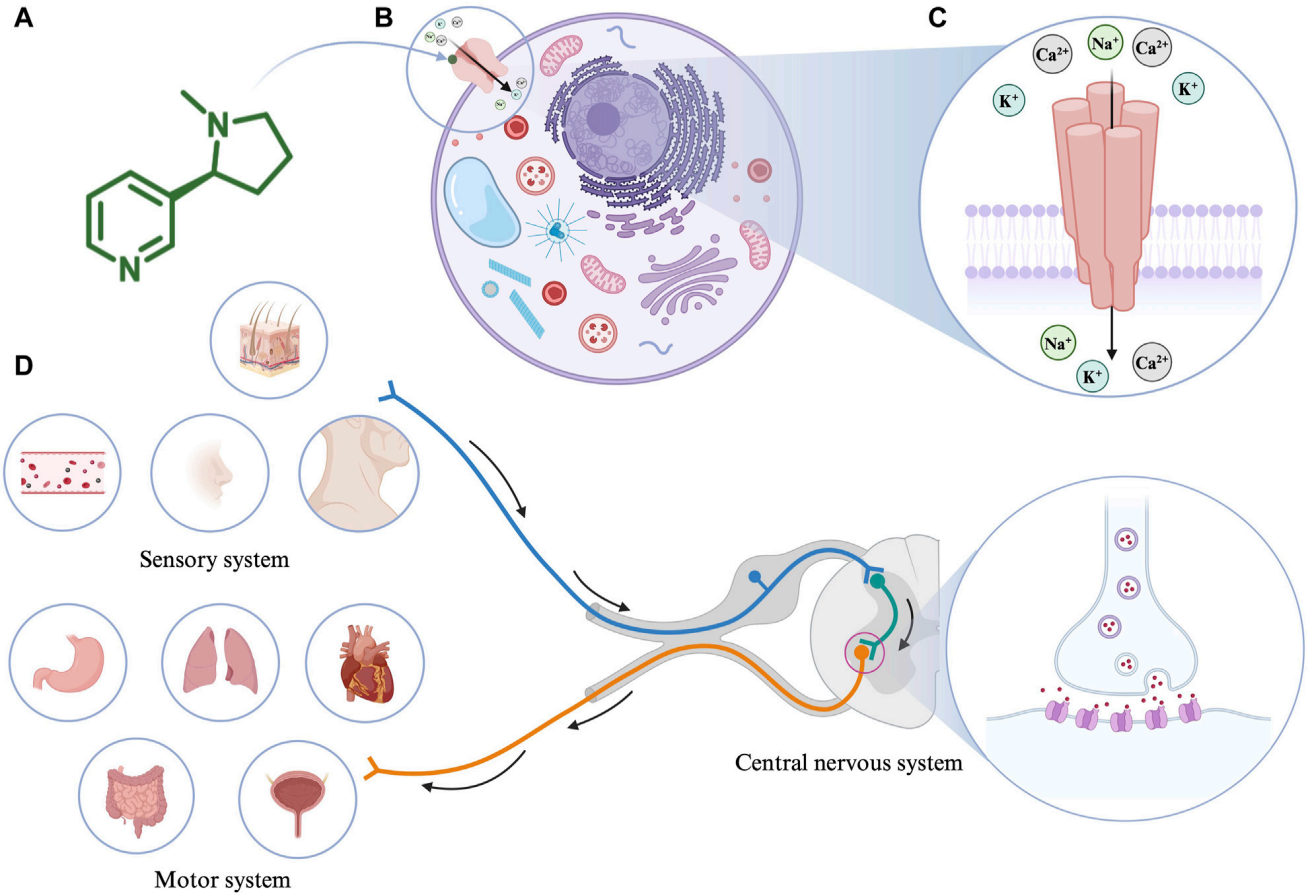
**(A)** The molecular structure of nicotine. **(B)** Interaction of nicotine with cells. **(C)** The structure of nicotinic acetylcholine receptor (nAChR). **(D)** The pharmacological effects of nicotine. Created with BioRender.com.

Nicotine (3-(1-methyl-2-pyrrolidinyl)-pyridine) chemically consists of a pyridine and pyrrolidine ring (Figure 1A). It is an important psychoactive substance and an addictive component of tobacco (Hussain et al., 2018).

Nicotine’s absorption is extremely fast and unmatched by any product used in nicotine replacement therapy (Benowitz et al., 1988). As an amphipathic organic base, when the pH value of the surrounding environment is appropriate, nicotine (pKa = 7.9) can directly pass through the cell membrane to alter mitochondrial respiration and the production of cell signaling molecules (Malińska et al., 2019). For example, nicotine is ionized and hydrophilic at low pH; therefore, it cannot pass through cell membranes. However, in the blood (pH = 7.4), 31% of nicotine is non-ionized and lipophilic, making it easy to cross the cell membrane and thus affecting the cell signal cascade (Yildiz, 2004). In the human body, the metabolic half-life of nicotine is 2 h. Consequently, 10%–20% of nicotine is directly excreted in the urine, and most of the remaining nicotine is metabolized by cytochrome P450 2A6 in the liver. Therefore, nicotine and its related products should be used cautiously in case of impaired liver function.

In addition, different nicotine elimination rates cause differences in plasma nicotine levels. Nicotine clearance rates can vary up to four times between individuals; therefore, different individuals will have different plasma and body nicotine levels when ingesting the same amount of nicotine. In addition, because nicotine is primarily metabolized in the liver, changes in hepatic blood flow, including exercise and meals, can affect the rate of nicotine elimination. Among these, meal consumption has been shown to affect blood nicotine levels 30 min after a meal. Therefore, pay particular attention to dietary effects when using nicotine products is essential.

In 1905, John Langley proposed that nicotine acts by binding to receptors (Langley, 1905). Notably, nAChRs, as the most critical nicotine acceptors, have been researched for a long time. As an agonist of nAChR, nicotine mediates various cellular processes by binding to nAChRs (Figure 1B). nAChRs are located in the plasma membrane and comprise five subunits that form homologous or heterologous pentamer. The subunits are organized around a central pore in the membrane (Figure 1C). They are expressed in the central and peripheral nervous systems (neuronal nAChRs), muscles (muscle nAChRs), and other tissues. Neuronal nAChRs include homopentamers composed of five identical α7, α8, or α9 subunits and heteropentamers comprising five α2-α6 or α10 subunits combined with β2-β4 subunits. Muscle nAChRs include heteropentamers composed of α1 subunits combined with β1, γ, δ, or ε subunits (Schuller, 2009). Different isoforms often play different roles in regulating physiological processes. When an agonist such as nicotine binds to nAChRs, the conformation of the receptor changes, the central ion channel opens, and cations, such as Na^+^, K^+^, and Ca^2+^, enter the cell from the outside (Gopalakrishnan et al., 1995; Lindstrom et al., 1996). The precise regulatory process of nAChR keeps it throughout the evolutionary process, among which α7nAChR and α4β2nAChR occupy dominant positions in the mammalian brain (Le Novère and Changeux, 1995). A variety of receptor agonists have been developed, such as the α4β2* nAChR agonists ABT-418, ispronicline and ABT-089 as well as the α7nAChR agonist GTS-21 (Sarter et al., 2009; Mei et al., 2018). Presently, most of the biological effects of nicotine are based on the research on α7nAChR and α4β2nAChR receptors. Nicotine appears to bind with higher affinity to α4β2nAChR than to α7nAChR (Gotti et al., 1997). The higher affinity results in long-term inactivation or desensitization of the α4β2nAChR when exposed to nicotine levels comparable to those in light or heavy smokers; however, the sensitivity of the α7nAChR is barely affected (Kawai and Berg, 2001).

The pharmacological effects of nicotine on the body are mainly categorized as the motor, sensory, and central nervous system responses (Figure 1D). In the motor system, low doses of nicotine activate the sympathetic ganglion cells. For example, nicotine acts on the heart, blood vessels, bladder, and gastrointestinal tract by stimulating the paraspinal sympathetic ganglia while increasing blood glucose levels and metabolic rates. Conversely, high-dose nicotine treatment causes these effects to disappear. Furthermore, nicotine causes twitching in skeletal muscles by acting on the motor endplates. Based on animal experiments, many effects of nicotine on the sensory system are associated with chemoreceptors. Low doses of nicotine initiate various bodily reflexes, including increased breathing rate and depth, vasoconstriction, increased heart rate, and increased blood pressure, by stimulating chemoreceptors in the carotid artery and aorta. In addition, low concentrations of nicotine can stimulate skin receptors through axonal reflexes, causing sweat secretion and hair erection (Comroe, 1960). In the central nervous system, nicotine acts mainly by binding to the presynaptic nAChR receptors. For example, nicotine promotes dopamine metabolism in the mesolimbic and nigrostriatal neurons by stimulating the presynaptic nAChR in dopaminergic neurons (Balfour, 1994).

## 3 Toxicities: the harmful effects of nicotine on the human body

**FIGURE 2:**
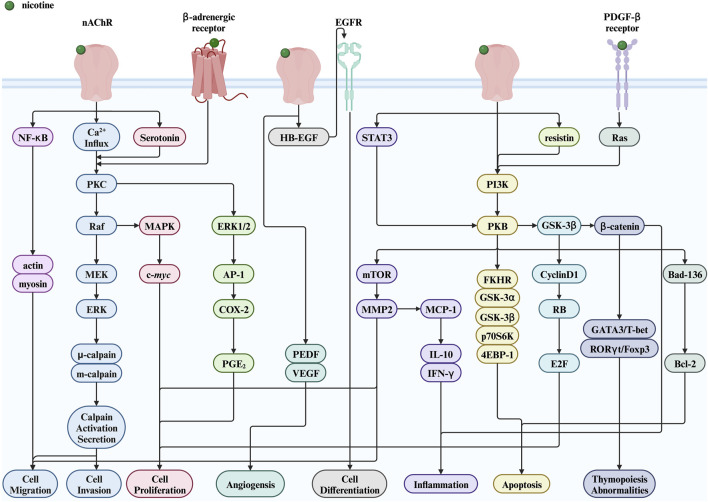
Nicotine toxicity and its related signaling pathways. Created with BioRender.com. (Abbreviations: MEK, MAPK/ERK kinase; PI3K, phosphatidylinositol-3-kinase; NF-κB, nuclear factor kappa-B; PKB, protein kinase B; GSK-3α, glycogen synthase kinase-3 alpha; GSK-3β, glycogen synthase kinase-3 beta; p70S6K, p70 ribosomal protein S6 kinase; 4EBP-1, a binding protein for eukaryotic translation initiation factor 4E; FKHR, a member of the forkhead transcription factor family; HB-EGF, heparin binding-epidermal growth factor; EGFR, epidermal growth factor receptor; PGE2, prostaglandin E2; STAT3, signal transducer and activator of transcription 3; RB, retinoblastoma protein; PEDF, pigment epithelium-derived factor; nAChR, nicotinic acetylcholine receptor; PKC, protein kinase C; ERK, extracellular signal-regulated kinase; MAPK, mitogen-activated protein kinase; AP-1, activator protein-1; COX-2, cyclooxygenase-2; mTOR, mechanistic target of rapamycin; MMP2, matrix metalloproteinase 2; MCP-1, monocyte chemoattractant protein-1; IL-10, interleukin-10; IFN-γ, interferon-γ; GSK-3β, glycogen synthase kinase-3 beta; E2F, early two factor).

This section focuses on the toxic nature of nicotine and introduces several diseases closely associated with nicotine use. Figure 2 shows a complete map of the molecular mechanisms that regulate various genes, transcription factors, and proteins involved in the pathogenic effects of nicotine. The descriptions explain the following five different types of diseases in detail.

### 3.1 Cancers

**FIGURE 3:**
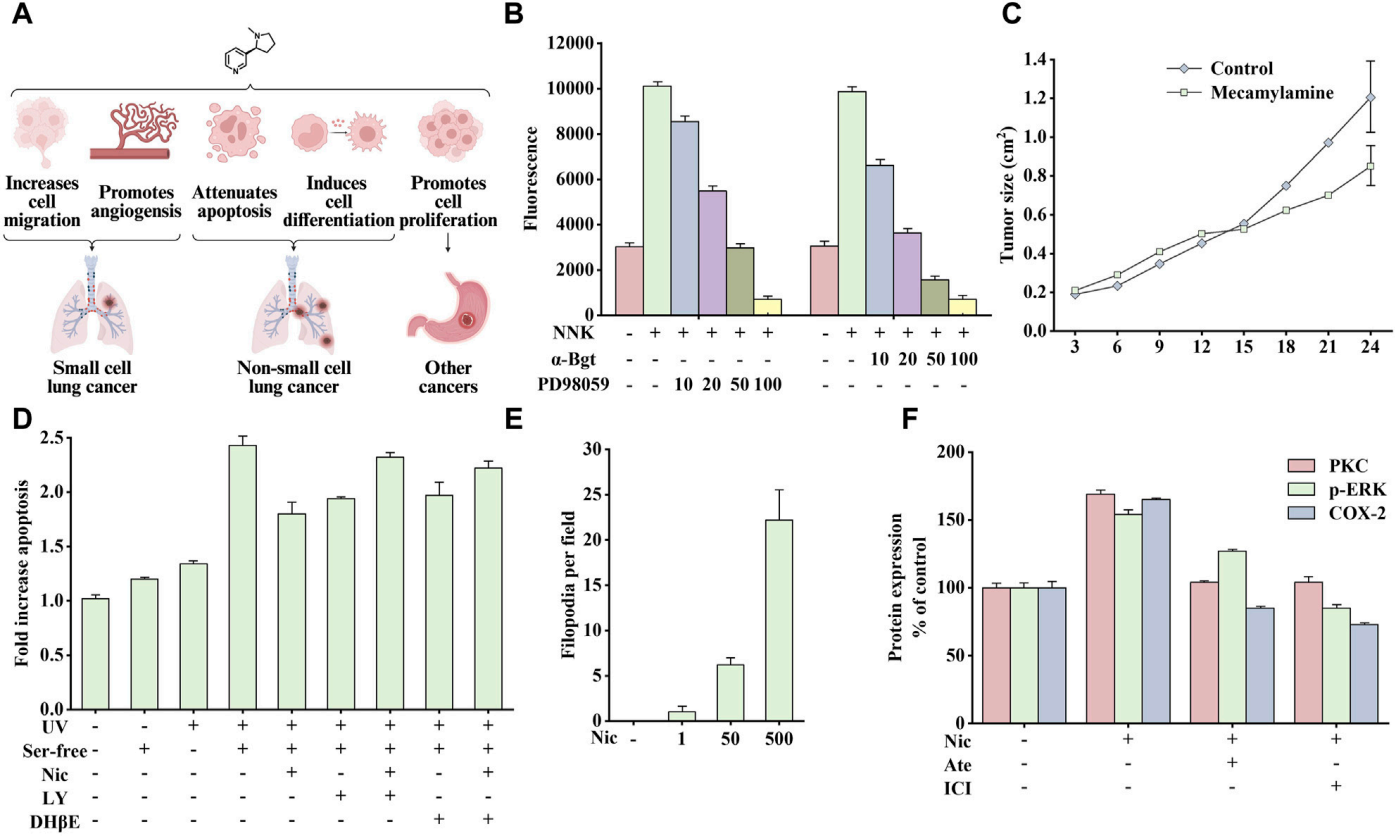
Effects of nicotine or 4-(methylnitrosamino)-1-(3-pyridyl)-1-butanone (NNK) on cancer and related data graphs. **(A)** The role of nicotine in cancer development. Created with BioRender.com. **(B)** The migration of cells under different treatment conditions. Reprinted (adapted) with permission from Ref. (Xu and Deng, 2004). Copyright 2004, Elsevier Inc. **(C)** The tumor growth of Lewis lung tumor model under mecamylamine treatment. Reprinted (adapted) with permission from Ref. (Heeschen et al., 2002). Copyright 2002, American Society for Clinical Investigation. **(D)** The apoptosis of cells under different treatment conditions. Reprinted (adapted) with permission from Ref. (West et al., 2003). Copyright 2003, American Society for Clinical Investigation. **(E)** The phenotypical changes of cells under nicotine treatment. Reprinted (adapted) with permission from Ref. (Martinez-Garcia et al., 2008). Copyright 2008, Elsevier Inc. **(F)** Protein kinase C (PKC), p-extracellular signal-regulated kinase (ERK), and cyclooxygenase-2 (COX-2) protein expression in nicotine, atenolol, and ICI 118551 treatment. Reprinted (adapted) with permission from Ref. (Shin et al., 2007). Copyright 2006, Oxford University Press. (Abbreviations: NNK, 4-(methylnitrosamino)-1-(3-pyridyl)-1-butanone; α-Bgt, α-bungarotoxin; Ser-free, serum-free; Nic, nicotine; LY, LY294002; DHβE, dihydro-β-erythroidine; Ate, atenolol; ICI, ICI 118551).

Cancer is one of the most widely discussed and recognized issues in biology and medicine. Its essence is the uncontrolled growth, division, and reproduction of a particular part of the cells in the human body, which destroys the body’s normal physiological functions, causes organ failure, and eventually leads to death. Studies have shown that nicotine is crucial in cancer induction, as shown in Figure 3A It promotes cell proliferation, inhibits apoptosis, and promotes phenotypic transformation, cell migration, and angiogenesis (Schuller, 2009).

#### 3.1.1 Small cell lung cancer

Small cell lung cancer (SCLC) is a typical neuroendocrine lung cancer. The influence of smoking on SCLC is particularly crucial. Notably, 4-(methylnitrosamino)-1-(3-pyridyl)-1-butanone (NNK), a nitrosation product of nicotine, has been identified as a potent carcinogen in tobacco smoke, and its affinity for α7nAChR is much higher than that of nicotine (Schuller and Orloff, 1998). Studies have shown that nicotine or NNK stimulates the release of autocrine growth factors serotonin and mammalian bombesin in a dose-dependent manner by binding to α7nAChRs (Cattaneo et al., 1993), which subsequently activates downstream protein kinase C (PKC)/Raf-1/mitogen-activated protein kinase (MAPK)/c-*Myc* mitogenic signaling pathway and promotes the growth of SCLC cell subpopulations *in vitro* (Jull et al., 2001). In addition, nicotine stimulates the release of serotonin and the proliferation of SCLC *in vitro*, which can be blocked by α-bungarotoxin (α-Bgt), indicating that α7nAChR is critical in the process of nicotine absorption, promoting the secretion of related endogenous neurotransmitters and mitosis (Codignola et al., 1994).

In addition, smoking promotes tumor production and induces cancer cell metastasis. The main members of the calpain family, μ-calpain and m-calpain, are widely expressed in SCLC cells. NNK activates ERK1 and ERK2 through α7nAChR/Ca^2+^ Influx/PKC/Raf/MAPK/ERK kinase (MEK) to phosphorylate μ-calpain and m-calpain, leading to calpain activation and secretion and promoting the migration and spread of cancer cells *in vivo*. As shown in Figure 3B, NNK significantly enhanced cell migration, which was blocked by PD98059 (inhibiting ERK1/2 activation) and α-Bgt, indicating that calpain is vital in this process (Xu and Deng, 2004). In addition, nicotine also promotes angiogenesis in various environments, such as inflammation, ischemia, and tumors, and this process is mainly mediated by α7nAChR. The stimulation of angiogenesis by α7nAChR is completely dependent on the phosphatidylinositol-3-kinase (PI3K)/MAPK/nuclear factor kappa-B (NF-κB) pathway and partially on vascular endothelial growth factor (VEGF). Therefore, pharmacological inhibition or genetic interference with α7nAChR expression can significantly reduce ischemia- and inflammation-induced angiogenesis. Similarly, inhibiting nAChR activity by mecamylamine can reduce angiogenesis and inhibit tumor growth (Figure 3C) (Heeschen et al., 2002).

#### 3.1.2 Non-small cell lung cancer

Non-small cell lung cancers include squamous cell carcinoma, lung adenocarcinoma, and large-cell carcinoma. Here, smoking is also a critical environmental factor. Squamous cell carcinoma mainly develops from bronchial epithelial cell lesions. In contrast, most lung adenocarcinomas arise from bronchiolar epithelial and alveolar type II cell lesions, in which both homomeric α7nAChRs and heteromeric α4β2nAChRs are present. However, exposure to nicotine concentrations comparable to those in smokers results in long-term inactivation of α4β2nAChRs, whereas α7nAChRs are largely unaffected (Kawai and Berg, 2001).

Studies on the above cells have shown that nicotine or NNK can rapidly activate PI3K/PKB (protein kinase B) to phosphorylate various downstream substrates, such as glycogen synthase kinase-3 alpha (GSK-3α), glycogen synthase kinase-3 beta (GSK-3β), p70 ribosomal protein S6 kinase (p70S6K), a binding protein for eukaryotic translation initiation factor 4E (4EBP-1), and a member of the forkhead transcription factor family (FKHR), ultimately inhibiting epithelial cell apoptosis. The above signal is mediated by nAChRs containing α3/α4 subunits (Brognard et al., 2001; West et al., 2003). As shown in Figure 3D, nicotine (10 µM) could prevent apoptosis induced by UV irradiation and serum-free culture. However, pretreatment with PI3K inhibitor LY294002 or α3/α4nAChR antagonist dihydro-β-erythroidine (DHβE) could reduce the anti-apoptotic effect of nicotine. Therefore, developing drugs that target the α3nAChR/PI3K/PKB signaling pathway may be promising for cancer treatment.

In addition, nicotine inhibits apoptosis induced by chemotherapeutic drugs. In three groups of human non-small cell lung cancers cells (A549, H23, and H1299), nicotine upregulated the X-linked inhibitor of apoptosis protein and survivin to inhibit apoptosis induced by gemcitabine, cisplatin, and taxol, which also depends on PKB regulation. When X-linked inhibitor of apoptosis protein and survivin are depleted, the inhibitory effect on apoptosis is lost (Dasgupta et al., 2006a). In another experiment, nicotine reduced serum deprivation or chemotherapy-induced apoptosis, which depends on NF-κB activation (Tsurutani et al., 2005).

In addition to inhibiting apoptosis, nicotine can promote cell proliferation through α7nAChR/β-arrestin/Src/Rb-Raf-1 pathway. Blocking either β-arrestin or Rb-Raf-1 interaction inhibits nicotine-induced proliferation (Dasgupta et al., 2006b). Consequently, nicotine can promote tumor development by inducing epithelial cell transformation. A study on normal bronchial epithelial cells showed that repeated stimulation with nicotine induces cells to differentiate into a neuron-like phenotype and secrete various neural cell adhesion molecules, thus enhancing intercellular adhesion after differentiation. Figure 3E shows the number of filamentous filopodia produced in normal human bronchial epithelial cells treated with different doses of nicotine. This process is associated with nicotine-mediated shedding of heparin binding epidermal growth factor (HB-EGF) and phosphorylation of the epidermal growth factor receptor (EGFR); while adding AG1478 (an inhibitor of EGFR tyrosine phosphorylation) can inhibit this effect (Martinez-Garcia et al., 2008).

#### 3.1.3 Other cancers

In addition to lung cancer, the harmful effects of nicotine on other cancers cannot be disregarded. Pancreatic ductal adenocarcinoma is an aggressive cancer closely associated with smoking, in which β-adrenergic receptors may be crucial. As a β1-and β2-AR agonist, NNK promotes the proliferation of pancreatic cancer cells in a hamster model. In immortalized human pancreatic cells, NNK activates β-adrenergic receptors, causing intracellular cyclic adenosine monophosphate (cAMP) accumulation and downstream phosphorylation of mitogen-activated protein kinase ERK1/2 to promote cell proliferation, which is inhibited by beta-blockers (propranolol), adenylyl cyclase inhibitors (SQ 22536) and Erk inhibitors (PD98059) (Askari et al., 2005). In the above experiment, NNK stimulated the proliferation through the cAMP/PKA/cAMP response element binding protein signaling cascade. However, γ-aminobutyric acid inhibits the isoproterenol-induced cAMP signaling through the γ-aminobutyric acid receptor. Therefore, stimulating γ-aminobutyric acid receptors may be useful in treating pancreatic cancer (Schuller et al., 2008). Further, nicotine also promotes pancreatic cancer development by stimulating the release of epinephrine and norepinephrine from the adrenal medulla (Li and Forsberg, 1996). Studies have shown that the migration and invasiveness of colon, prostate, and breast cancers are associated with β-adrenergic activity (Masur et al., 2001; Drell et al., 2003; Palm et al., 2006).

A study using HT-29 cells identified the mechanism that nicotine promotes the growth of colon cancer cells. Nicotine increases the expression of catecholamine synthases tyrosine hydroxylase (TH), dopamine-β-hydroxylase, and phenylethanolamine N-methyltransferase in a dose-dependent manner, thereby promoting adrenaline synthesis. Adrenaline stimulates colon cancer cell proliferation by acting on adrenoceptors, and α7nAChRs are involved in this process. Methyllycaconitinee, an α7nAChR antagonist, reversed the stimulatory effects of nicotine on cell proliferation, TH and dopamine-β-hydroxylase expression, and adrenaline production (Wong et al., 2007).

β-adrenergic receptors were also crucial in researching gastric cancer development with nicotine use. In Shin’s study, nicotine stimulated β-adrenergic receptors and subsequently activated the downstream PKC/ERK1/2/activator protein-1 (AP-1)/cyclooxygenase-2 (COX-2)/prostaglandin E2 (PGE2) signaling cascade to promote gastric cancer cell growth and proliferation (Shin et al., 2007). As shown in Figure 3F, atenolol and ICI 118551 (β1 and β2-AR antagonists, respectively) reversed PKC expression, ERK1/2 phosphorylation, and COX-2 upregulation induced by nicotine. In another study, by orthotopically transplanting gastric cancer cells into the stomach wall of nude mice, the tumor area was found to be significantly larger than that in the control group after 3 months of nicotine treatment; which was associated with the ERK/COX-2/VEGF signaling pathway (Shin et al., 2004).

### 3.2 Cardiovascular diseases

Presently, cardiovascular diseases have become the leading cause of death in developed and developing countries, and the global death toll increased from 12.1 million in 1990 to 18.6 million in 2019 (Roth et al., 2020). As the population ages, the death toll is also expected to rise.

**FIGURE 4:**
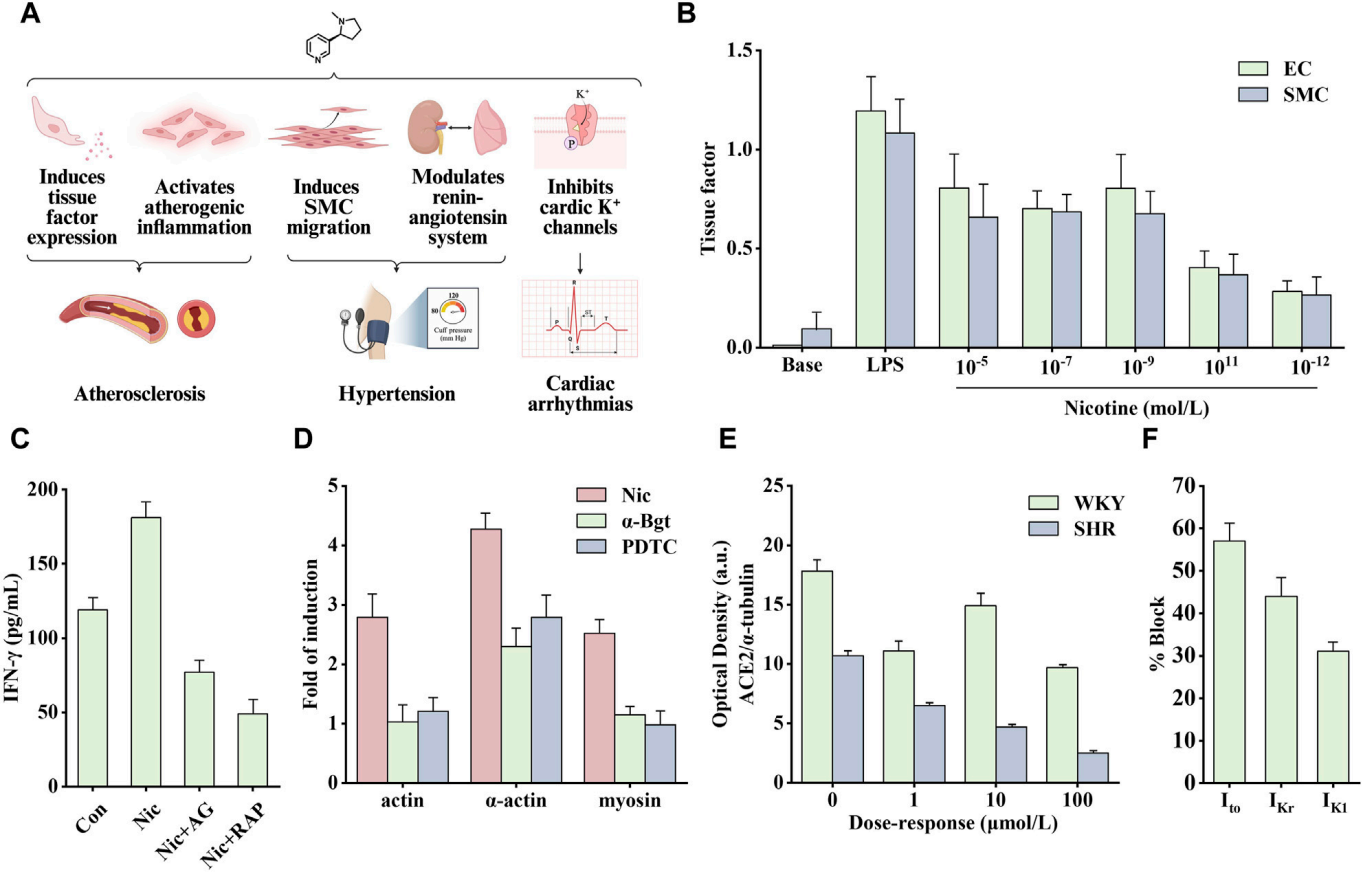
Effects of nicotine on cardiovascular diseases and related data graphs. **(A)** The effects of nicotine on cardiovascular diseases. Created with BioRender.com. **(B)** The tissue factor in endothelial cells (EC) and smooth muscle cells (SMC) under lipopolysaccharide (LPS) or nicotine treatment. Reprinted (adapted) with permission from Ref. (Cirillo et al., 2006). Copyright 2006, Elsevier Inc. **(C)** Interferon-γ (IFN-γ) released by cells under nicotine, AG490, and rapamycin treatment. Reprinted (adapted) with permission from Ref. (Xu et al., 2019). Copyright 2006, Xu et al. **(D)** Cell cytoskeletal protein expression under nicotine, α-bungarotoxin, and PDTC treatment. Reprinted (adapted) with permission from Ref. (Wang et al., 2013). Copyright 2012, Elsevier Ltd. **(E)** Angiotensin-converting enzyme 2 (ACE2) protein expression in spontaneously hypertensive (SHR) and Wistar Kyoto (WKY) rats under nicotine treatment. Reprinted (adapted) with permission from Ref. (Ferrari et al., 2007). Copyright 2007, Springer Nature. **(F)** K+ currents in ventricular myocytes under nicotine treatment. Reprinted (adapted) with permission from Ref. (Wang et al., 1999). Copyright 1999, Elsevier Inc. (Abbreviations: SMCs, smooth muscle cells; Nic, nicotine; AG, AG490; RAP, rapamycin; α-Bgt, α-bungarotoxin; Ito, transient outward K+ current; IKr, delayed rectifier K+ current; IK1, inward rectifier K+ current).

Cardiovascular diseases affect the heart and blood vessels (arteries, veins, and capillaries). They can be divided into acute and chronic diseases and are generally associated with arteriosclerosis. Notably, several causes of cardiovascular disease include poor eating habits, lack of exercise, smoking, and drinking. Among them, smoking is considered the main preventable factor (Roth et al., 2020). As the main particle phase component of smoke produced by tobacco combustion (Centner et al., 2020), the pathogenic effect of nicotine has also been extensively studied (Figure 4A).

#### 3.2.1 Cardiovascular diseases

Atherosclerosis mainly occurs in the coronary arteries, cerebral arteries, and aorta and is the pathological basis of many cardiovascular diseases. After the endothelial cells are damaged, they capture adhesion molecules, attract lymphocytes and monocytes to infiltrate the arterial wall and induce inflammation. White blood cells, fat, and cholesterol floating in the blood vessels are then deposited, resulting in plaque formation, stiffness, and thickening of the arterial inner wall (Tabas et al., 2015). Once the plaque ruptures, platelets accumulate at the top, plugging the already narrowed lumen and restricting oxygen and nutrient uptake by the associated cells. If this process occurs in the blood vessels of the heart, it causes myocardial infarction and death of heart muscle cells that do not receive nutrients. Stroke occurs when an embolism blocks the blood flow to the brain. During this process, there is increased proliferation and migration of vascular smooth muscle cells (VSMCs). Nicotine is involved in almost all stages of atherosclerosis occurrence and development.

Nicotine promotes the secretion of various cytokines, such as basic fibroblast growth factor, transforming growth factor beta, VEGF, and platelet-derived growth factor (PDGF), stimulating the vascular growth process (Grozio et al., 2007). A study on VSMCs found that nicotine modulates aortic production of basic fibroblast growth factor and transforming growth factor beta, which is critical in developing neointimal fibroplasia (Cucina et al., 2000). In addition, Carty et al. found that in human smooth muscle cells (SMCs), nicotine and its metabolite cotinine promoted the production and secretion of basic fibroblast growth factor and upregulated the expression of several matrix metalloproteinases (MMPs), such as collagenase-1, stromelysin-1 and gelatinase A, which are crucial in cell migration (Carty et al., 1996). When exposed to nicotine, the expression of tissue factor, a small molecular glycoprotein involved in the regulation of blood coagulation, hemostasis, and thrombus formation, is significantly increased in endothelial cells (ECs) and SMCs (Figure 4B). This process is regulated by the transcription factor NF-κB (Cirillo et al., 2006). Tissue factor forms a tissue factor-Vlla complex with coagulation factor Vlla to activate factors IX and X to generate thrombin (Toschi et al., 1997). To sum up, nicotine regulates the secretion of various growth factors and promotes the development of atherosclerosis.

Therefore, the role of inflammation in disease development should not be underestimated. Nicotine binds to nAChR to promote the opening of ion channels and regulate intracellular Na^+^, K^+^, Ca^2+^, and other ion concentrations, thereby activating different signaling cascade pathways and promoting atherosclerotic inflammation. Among them, the signal transducer and activator of transcription 3 (STAT3) is an important transcription factor involved in regulating various extracellular signals associated with cell growth and inflammation. After STAT3 activation, it enters the nucleus to promote the transcription of target genes. Studies have shown that there is a direct interaction between STAT3 and α1nAChR. After knocking out α1nAChR, the upregulation of p-STAT3, p-PKB, and p-mechanistic target of rapamycin (mTOR) induced by nicotine *in vitro* was significantly reduced, whereas the effect was opposite when α1nAChR was overexpressed (Xu et al., 2019). Staining for activated STAT3 in sections of human atherosclerotic lesions showed that the nuclei of cells in areas of inflammation stained strongly, in contrast to those of cells in areas with little or no inflammation. Endothelial STAT3 knock-out mice have smaller atherosclerotic lesions, and this inhibition may involve the PKB/mTOR/MMP2 signaling pathway. As shown in Figure 4C, nicotine upregulated levels of the pro-inflammatory factor interferon-γ (IFN-γ), which was inhibited by AG490 (STAT3 inhibitor) and rapamycin (mTOR inhibitor) (Gharavi et al., 2007; Xu et al., 2019). In another study on VSMCs, nicotine upregulated the production of reactive oxygen species (ROS). It activated the pattern recognition NOD-like receptor thermal protein domain associated protein 3 (NLRP3). NLRP3 activation leads to elevated C-reactive protein levels, an inflammatory cytokine that induces vascular inflammation. In addition, pro-caspase-1 formed inflammasomes by combining with NLRP3, simultaneously releasing inflammatory factors interleukin (IL)-18 and IL-1β, thus aggravating the inflammatory response (Hecker et al., 2015; Yao et al., 2019). By treating human aortic endothelial cells with nicotine, the above signaling pathway was found to mediate pyroptosis, and the nicotine-NLRP3-apoptosis-associated speck-like protein containing CARD (ASC)-pyroptosis pathway was activated by ROS (Wu et al., 2018).

Endothelial dysfunction and altered vascular smooth muscle function are involved in the progression of atherosclerosis. Reduced NO production is a typical feature of endothelial dysfunction. Nicotine causes significant structural and functional changes in the aorta. The combination of nicotine and nAChR promoted the activation of ERK1/2 and activated NF-κB, which stimulated the synthesis of intercellular adhesion molecule one and vascular cell adhesion molecule 1 (Egleton et al., 2009). In addition, nicotine can also stimulate macrophages to secrete tumor necrosis factor alpha (TNF-α) and IL-1β, increase the expression of human EC adhesion molecules, and lead to increased ahesion of monocytes to human umbilical vein ECs (Wang Y. et al., 2004), which may be one of the causes of EC disorders. In addition, Wang et al. found that nicotine-induced autophagy promoted VSMC phenotypic transformation and is partially mediated through the nAChRs/ROS/NF-κB signaling pathway (Wang Y. et al., 2004). NF-κB was also involved in the upregulation of nicotine-induced cytoskeletal proteins through α7nAChR. As shown in Figure 4D, the α7nAChR inhibitor α-Bgt and the NF-κB inhibitor PDTC significantly inhibited the expression of nicotine-induced cytoskeletal proteins, including actin, α-actin, and myosin (Wang et al., 2013). Another study showed that nicotine activated the death-associated protein kinase 3/AMP-activated protein kinase (AMPK) signaling cascade through receptors in VSMCs, thereby inducing endoplasmic reticulum stress-related protein expression and VSMC differentiation (Li K. X. et al., 2019).

#### 3.2.2 Hypertension and cardiac arrhythmias

In addition to atherosclerosis, it seems that nicotine is more than just a bystander of hypertension or arrhythmias. Nicotine alters vascular tone by modulating the release of NO, bradykinin, and leukotrienes (Kuhlmann et al., 2005). In addition, nicotine acts as a sympathomimetic agent to increase blood pressure by stimulating the release of catecholamines (Benowitz, 2001). Angiotensin-converting enzyme 2 (ACE2) can convert angiotensin II to angiotensin (1–7) to promote vasodilation and inhibit proliferation. Nicotine inhibits this process by reducing ACE2 expression, indirectly leading to increased blood pressure. As shown in Figure 4E, nicotine dose-dependently reduced the optical density of ACE2 protein in cultured neurons of spontaneously hypertensive and Wistar Kyoto rats (Ferrari et al., 2007).

Arrhythmias include atrial and fibrillation, ventricular fibrillation, and tachycardia. Nicotine can induce arrhythmias by increasing the automaticity of the sinoatrial node and accelerating conduction through the atrioventricular node (Benowitz and Gourlay, 1997), which may involve multiple factors. Studies have shown that nicotine increases catecholamine release by stimulating β1 receptors and stimulates sympathetic nerve activity to increase heart rate and blood pressure through direct peripheral and centrally mediated effects (Shinozaki et al., 2008). In addition, the increase in heart rate may be associated with a decreased vagal tone (Benowitz and Gourlay, 1997). The cellular mechanism involved may be related to the function of nicotine in prolonging action potential and depolarizing membrane potential. As a non-specific blocker of K^+^ channel, nicotine directly inhibits cardiac K^+^ channels to block various types of K^+^ currents, including transient outward K^+^ current (Ito), delayed rectifier K^+^ current (I_Kr_), and inward rectifier K^+^ current (I_K1_) (Figure 4F) (Wang et al., 1999). Therefore, nicotine likely causes arrhythmias and heart problems by inhibiting cardiac K^+^ channels.

### 3.3 Respiratory diseases

**FIGURE 5:**
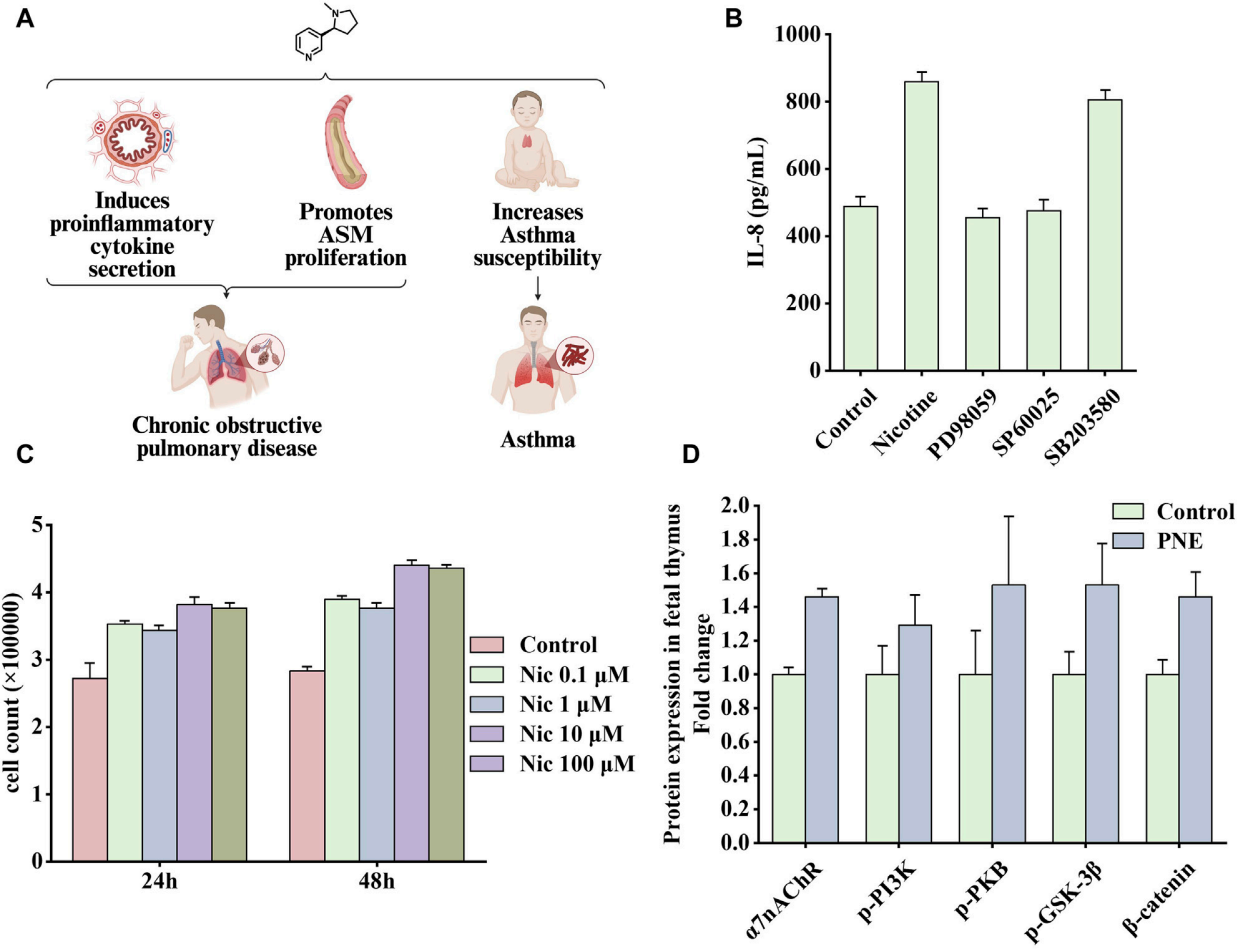
Effects of nicotine on respiratory diseases and related data graphs. **(A)** The effects of nicotine on respiratory diseases. Created with BioRender.com. **(B)** Interleukin-8 (IL-8) protein concentration in cells under nicotine, PD98059, SP60025, and SB203580 treatment. Reprinted (adapted) with permission from Ref. (Tsai et al., 2006). Copyright, 2006, Mary Ann Liebert, Inc. **(C)** Cell numbers under nicotine treatment. Reprinted (adapted) with permission from (He et al., 2014). Copyright, 2014, Public Library Science. **(D)** Effects of prenatal nicotine exposure (PNE) on protein expression of α7 nicotinic acetylcholine receptor (α7nAChR), p-phosphatidylinositol-3-kinase (PI3K), p-protein kinase B (PKB), p-glycogen synthase kinase three beta (GSK-3β), and β-catenin during fetal thymopoiesis. Reprinted (adapted) with permission from Ref. (Wen et al., 2022). Copyright, 2022, Elsevier Inc. (Abbreviations: ASM, airway smooth muscle; Nic, nicotine).

Respiratory diseases are common and frequently occurring conditions. The primary lesions are located in the trachea, bronchi, lungs, and chest cavity. Patients with mild symptoms often experience cough, chest pain, and altered breathing. Patients with severe symptoms experience dyspnea, hypoxia, and even death due to respiratory failure. Two diseases are highlighted in this summary: chronic obstructive pulmonary disease (COPD) and tuberculosis, in which smoking is an important pathogenic environmental factor. Figure 5A shows the effects of nicotine on these diseases.

#### 3.3.1 Chronic obstructive pulmonary disease

COPD is a classic lung disease characterized by persistent airflow limitation. It is often associated with an increased chronic inflammatory response of the airways and lungs to noxious particles or gases, ultimately leading to accelerated aging of the lungs. It includes chronic obstructive bronchitis (narrowing of the small airways) and emphysema (damage to the alveoli). In the lungs of healthy individuals, alveoli attach to the small airways to keep them open, whereas in patients with COPD, the peripheral wall of the bronchioles is thickened, small airways are narrowed, alveoli are damaged, and mucus production increases (Christenson et al., 2022). Smoking is the main risk factor for developing COPD. Therefore, understanding its physiological mechanism is essential for the treatment and prevention of the disease.

Smoking causes small airway remodeling. In a guinea pig model, exposure to cigarette smoke produced symptoms similar to small airway remodeling, such as increased airway resistance, decreased airflow, and air trapping (Wright et al., 2007). A study showed that nicotine promoted the proliferation of human airway smooth muscle cells, and this process was associated with the α5nAChR-mediated Ca^2+^ influx dependent on transient receptor potential canonicals (TRPCs) channels. Knocking out the TRPC3 gene attenuated nicotine-induced Ca2^+^ influx and cell proliferation effects (Jiang et al., 2019). Another study explored the impact of nicotine addiction on the expression of 1800 genes using microarray bioinformatics analysis. Through integration, several overexpressed genes were found to be involved in the MAPK pathway, among which ERK1/2 and c-Jun N-terminal kinase (JNK) were more likely to function (Tsai et al., 2006). In addition, the MAPK pathway mediates IL-8 production. As an important pro-inflammatory factor, IL-8 may be associated with lung inflammation and tumorigenesis. As shown in Figure 5B, nicotine increased the synthesis of IL-8, which was inhibited by PD98059 (ERK 1/2 inhibitor) or SP600125 (JNK inhibitor); however, the inhibitor of ribonuclease P protein subunit p38 (p38) SB203580 had little effect on this process. Another characteristic of COPD is increased airway smooth muscle mass, in which PKB plays an important role. PKB, also known as protein kinase B, as an essential signaling hub, participates in various signaling pathways related to cell proliferation, apoptosis, and migration (Liang and Slingerland, 2003). In a vitro rat airway smooth muscle cell model, nicotine bound to nAChRs and subsequently activated the PI3K/PKB/GSK-3β/cyclin D1/retinoblastoma protein (RB)/early two factor (E2F) signaling cascade, ultimately leading to a significant increase in DNA synthesis and cell number. As shown in Figure 5C, nicotine treatment at concentrations of 0.1, 1, 10, and 100 μM significantly increased the number of cells (He et al., 2014). In addition, studies have shown that patients with COPD have a significantly increased risk of developing SCLC (Purdue et al., 2007). In this experiment, mice with COPD were more prone to develop neuroendocrine tumors after exposure to nicotine, whereas healthy mice in the control group had no tumors (Schuller et al., 1995).

#### 3.3.2 Asthma

Asthma is an inflammatory disease of the lungs characterized by hyperresponsiveness and reversible obstruction of the large and small conducting airways. Globally, more than 300 million individuals are affected by asthma (Holgate et al., 2015).

In several animal studies, Holgate et al. found that prenatal exposure to nicotine increased the probability of asthma in the offspring. This may be associated with the downregulation of homeostatic lung mesenchymal peroxisome proliferator-activated receptor γ (PPARγ) signaling, which mediates paracrine communication of specific molecules between the alveolar epithelium and interstitium. Nicotine treatment in pregnant rats showed that PPARγ was downregulated, and the expression of mesenchymal markers of tracheal contraction response and airway contraction was significantly increased. However, the PPARγ agonist, rosiglitazone, could effectively block the above changes (Holgate et al., 2015). In addition, nicotine promotes the trans differentiation of alveolar interstitial fibroblasts to myofibroblasts. Alveolar interstitial fibroblasts convert to a phenotype detrimental to alveolar homeostasis, resulting in damage to developing alveoli and lung injury. However, upregulation of PPARγ can block this transformation (Krebs et al., 2010). The above experiments suggest that PPARγ may serve as a novel target for developing asthma drugs.

Another study identified signaling pathways associated with asthma susceptibility in mice (Wen et al., 2022). By administering 3 mg/kg/day of nicotine to establish a prenatal nicotine exposure (PNE) mouse model, this study showed that PNE impaired fetal thymus and postnatal CD4^+^ T cell development. Figure 5D shows that during fetal thymogenesis, nicotine treatment upregulated the expression of α7nAChR and subsequently increased the phosphorylation level of downstream PI3K/PKB/GSK-3β signaling molecules, which inhibited the process in which GSK3β degraded β-catenin and increased the levels of β-catenin. In addition, it seems that increased β-catenin level causes the fetal thymus to shape a Th2/Th17 bias-generating gene expression pattern during generation, characterized by higher expression levels of GATA3/T-bet and RORγt/Foxp3, thus leading to thymopoiesis abnormalities.

### 3.4 Reproductive system diseases

**FIGURE 6:**
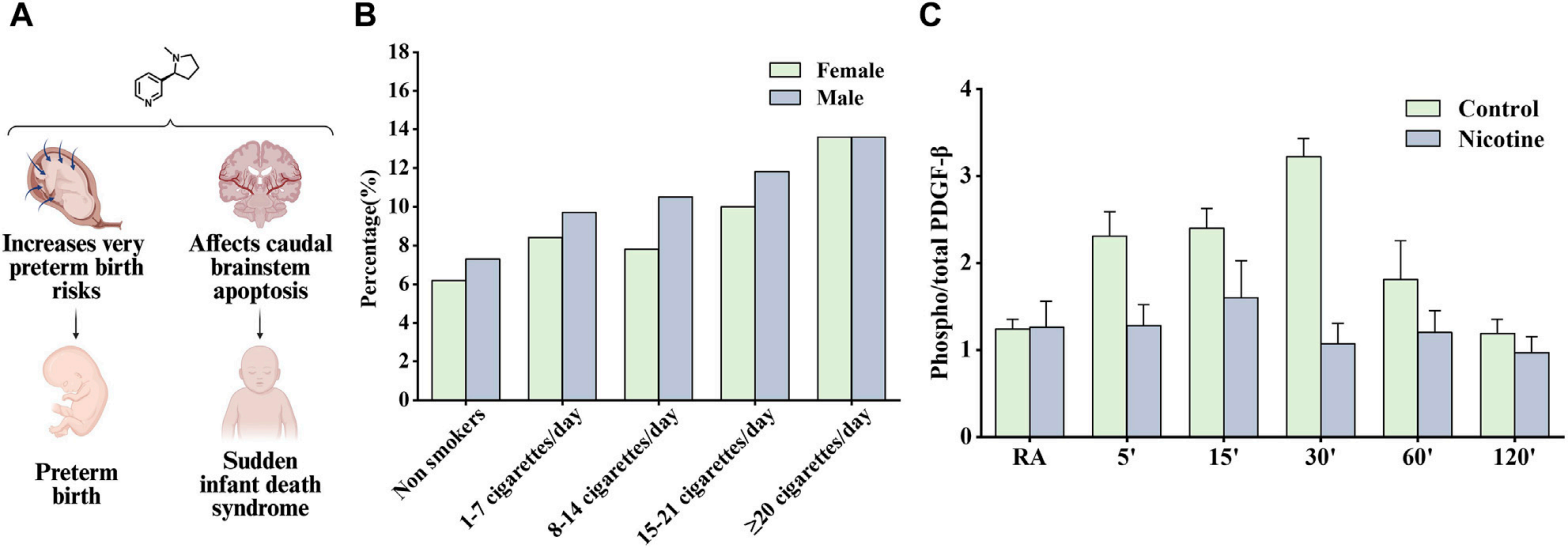
Effects of nicotine on reproductive diseases and related data graphs. **(A)** The effects of nicotine on reproductive system diseases. Created with BioRender.com. **(B)** Impact of fetal gender in combination with maternal smoking on preterm birth. Reprinted (adapted) with permission from Ref. (Günther et al., 2021). Copyright, 2020, Cambridge University Press. **(C)** Effects of prenatal nicotine exposure (PNE) on the phosphorylation of platelet-derived growth factor (PDGF)-β receptor during hypoxia. Reprinted (adapted) with permission from Ref (Simakajornboon et al., 2010). Copyright, 2010, Elsevier Ireland Ltd. (Abbreviations: RA, room air).

Studies have shown that tobacco exposure before and during pregnancy can lead to adverse outcomes, such as reduced fertility and increased maternal, fetal, and infant morbidity and mortality, further aggravating the adverse effects on the mother and offspring, in which nicotine, the main ingredient of tobacco, seems to play an important role (National Center for Chronic Disease et al., 2014). Figure 6A shows some influence of nicotine on preterm birth and sudden infant death syndrome.

#### 3.4.1 Preterm birth

Several studies have suggested that nicotine is contributed to the increased risk of preterm birth associated with smoking. Schuller et al. found that smoking increased the risk of preterm birth due to premature rupture of membranes and bleeding in late pregnancy (Kyrklund-Blomberg et al., 2005). Günther et al. found that all smoking groups had the higher preterm birth rate compared to nonsmokers and the majority of preterm infants were male except for very heavy smokers (≥22 cigarettes/day) (Figure 6B). Women who used snus and continued smoking had an increased risk of preterm birth compared with women who had quit smoking, as tested using a multiple logistic regression model. The nicotine content in snus is similar to that absorbed from cigarettes but contains no other ingredients, clarifying the role of nicotine in inducing preterm birth (Baba et al., 2012). A subsequent study recommended a reduction in nicotine use during pregnancy and showed that snus was associated with preterm birth. The study also demonstrated that smoking cessation before antenatal appointments was not associated with an increased risk of preterm birth (Dahlin et al., 2016).

#### 3.4.2 Sudden infant death syndrome

Sudden infant death syndrome is an important cause of death in infants aged 1 month to 1 year; however, its etiology is unknown and may be associated with impaired cardiorespiratory control and arousal responses, but perinatal exposure to cigarette smoke has been listed as a risk factor (Mitchell and Milerad, 2006). Studies have shown that nicotine can accumulate in breast milk, placenta, and amniotic fluid, continue to have adverse effects on fetuses and newborns, and may involve the interaction of cellular mechanisms such as oxidative stress, inflammation, and endoplasmic reticulum stress (Wong et al., 2015). Infants who died of sudden infant death syndrome often experienced severe bradycardia and apnea, and Huang et al. considered the above responses as cardiorespiratory hyperresponses to hypoxia or hypercapnia. Subsequent findings in hypoxic/hypercapnic mice show that prenatal exposure to nicotine recruits excitatory neurotransmission to cardiac vagal neurons, leading to heart rate changes (Huang et al., 2005). In another study, α4nAChRs in the preBötzinger Complex were found to regulate glutamatergic neurotransmitter transmission and respiratory rate, and activating α4nAChRs on sublingual (XII) motor neurons was reported to increase respiratory rate, which may also be the pharmacological basis of nicotine contributing to sudden infant death syndrome development (Shao and Feldman, 2009). However, Simakajornboon et al. suggested a different point of view that the hypoxic ventilatory response may be associated with the PDGF-β receptor in the caudal brainstem and its downstream anti-apoptotic cascade. In a rat model, prenatal nicotine exposure attenuated the phosphorylation of the PDGF-β receptor (Figure 6C). Subsequently, it activated the Ras/PI3K/PKB/Bad-136/Bal-2/RB/E2F signaling cascade during hypoxia in the developing rat caudal brainstem, which is thought to increase caudal brainstem cell apoptosis and the vulnerability of neuronal cells in the respiratory control zone (Simakajornboon et al., 2010).

### 3.5 Periodontitis and age-related macular degeneration

**FIGURE 7:**
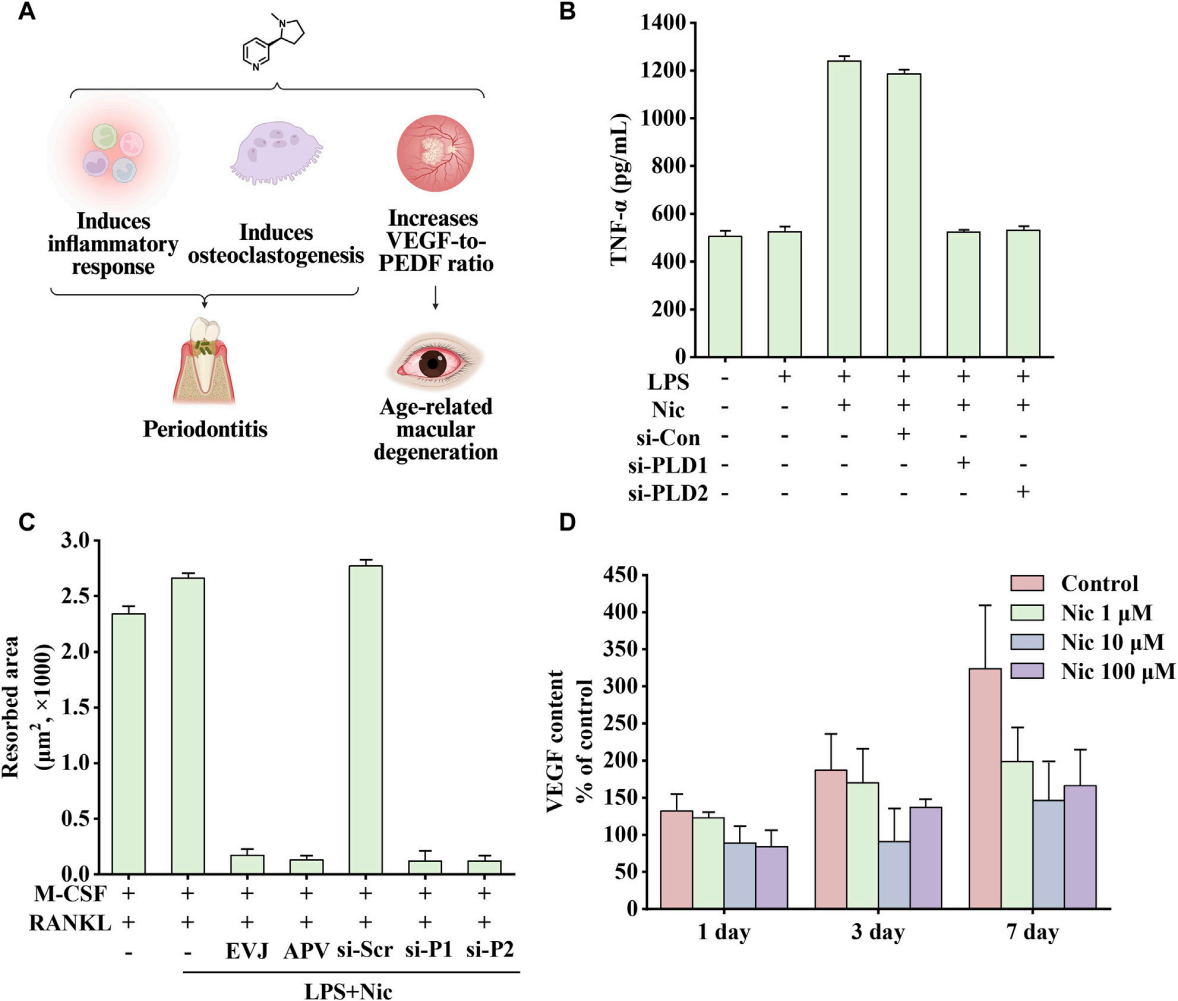
Effects of nicotine on periodontitis and age-related macular degeneration (AMD) and related data graphs. **(A)** The effects of nicotine on periodontitis and AMD. Created with BioRender.com. **(B)** Secretion of tumor necrosis factor-alpha (TNF-α) in cells under different treatments. Reprinted (adapted) with permission from Ref. (Shin et al., 2015). Copyright, 2015, Wiley-VCH. **(C)** The area of osteoclast-induced resorption under different treatments. Reprinted (adapted) with permission from Ref. (Shin et al., 2015). Copyright, 2015, Wiley-VCH. **(D)** Vascular endothelial growth factor (VEGF) content in cells under nicotine treatment. Reprinted (adapted) with permission from Ref. (Klettner et al., 2012). Copyright, 2012, Springer Nature. (Abbreviations: PEDF, pigment epithelium-derived factor; LPS, lipopolysaccharide; Nic, nicotine; si-Con, Control siRNA; si-PLD1, phospholipase D1 (PLD1) siRNA; si-PLD2, phospholipase D1 (PLD2) siRNA; RANKL, receptor activator of nuclear factor kappa-B ligand).

In addition to its involvement in the development of aforementioned diseases, nicotine plays a pro-inflammatory role in many other diseases, such as periodontitis and age-related macular degeneration (AMD). Figure 7A shows some effects of nicotine on periodontitis and AMD.

#### 3.5.1 Periodontitis

Periodontitis, the leading cause of tooth loss in adults, is the result of dysbiosis of the oral microbiota, which interacts with host defense mechanisms (Kinane et al., 2017).

Inflammatory response is critical in disease development. Phospholipase D (PLD), including two subtypes, PLD1 and PLD2, is a signal transduction enzyme. It is expressed in almost all mammalian tissues and involves in various physiological functions, including lipid degradation, cell proliferation, cell differentiation, and immune response process (Locati et al., 2001; Jenkins and Frohman, 2005). Notably, compared with healthy controls and non-smoking patients, mRNA expression of PLD1 and PLD2 was significantly increased in smoking patients, which is consistent with the upregulation in HPDLC stimulated by nicotine and lipopolysaccharide (LPS) *in vitro* (Shin et al., 2015). In addition, inflammatory factors TNF-α and IL-1β can significantly upregulate the expression of PLD, and perhaps the overall increase in inflammatory response is responsible for promoting PLD expression. NO, PGE2, IL-1β, TNF-α, and IL-8 are critical in the occurrence and development of periodontitis (Agarwal et al., 1995). Therefore, blocking the expression of PLD can inhibit nicotine and LPS-induced changes in the abovementioned factors, which is consistent with the findings of previous studies. As shown in Figure 7B, PLD1 and PLD2 siRNA attenuates nicotine and LPS-induced TNF-α expression (Sethu et al., 2010; Kang et al., 2013; Sethu, 2013).

In Kang’s study, nicotine increased β-catenin levels and inflammation through the resistin/PI3K/PKB/GSK3β signaling cascade. In addition, by injecting nicotine at a dose of 0.7 mg/kg in rats for 30 days, Li et al. found that the above treatment would reduce the levels of bone alkaline phosphatase and osteocalcin, increase the expression of TNF-α and COX-2, and increase alveolar bone loss (Li et al., 2017). In addition, nicotine and LPS activate the PI3K/PKC/MAPK pathway to promote the expression of inflammatory factors and mediate the development of osteoclasts through the NF-κB/c-Fos/NFCTc1 signaling pathway in human periodontal ligament cells. Figure 7C shows that nicotine increased the area of osteoclast-induced absorption pits, which were eliminated by EVJ, APV, PLD1 siRNA, and PLD2 siRNA (inhibiting PLD isoforms) (Shin et al., 2015).

As a key regulator in osteoarthritis (OA) development, hypoxia-inducible factor-2α (HIF-2α) was increased in periodontal ligament cells of patients with periodontitis. Bae et al. found that combined treatment with nicotine and LPS induced the production of NO and PGE2, and upregulated the expression of inducible nitric oxide synthase (iNOS), COX-2 protein in human periodontal ligament cells. Besides, the mRNA expression of various pro-inflammatory cytokines such as TNF-α, IL-1β, IL-6, IL-8, IL-10, IL-11, and IL-17 was also upregulated. In addition, the expression of collagenases (MMP1, MMP8, and MMP13) and gelatinases (MMP2 and MMP9) was upregulated, and various cytokines, including PKB, Janus kinase 2 (JAK2), STAT3, ERK, and JNK-MAPK, were activated. Moreover, an increase in the number of osteoclasts and osteoclast-specific genes was also observed. These effects were attenuated by HIF-2α inhibitor or HIF-2α siRNA. Therefore, inhibiting HIF-2α inhibits inflammatory cytokines and blocks osteoclast differentiation (Bae et al., 2015). Interestingly, another study by Cho et al. showed that inhibition or silencing of peptidyl-prolyl cis/trans isomerase NIMA-interacting protein 1 (PIN1) played a similar role. Inhibiting PIN1 by juglone or the knockdown of PIN1 gene expression by siRNA attenuated nicotine and LPS-induced PGE2, NO production, COX-2 and iNOS expression, NF-κB activation, increased osteoclast number, and osteoclast-specific gene expression, whereas overexpression of PINI enhanced these effects (Cho et al., 2015).

#### 3.5.2 Age-related macular degeneration

AMD is the leading cause of vision loss in people aged >55 years in developed countries and is expected to affect 288 million people worldwide by 2040. The pathology of AMD is characterized by massive accumulation of extracellular deposits that form drusen (Fleckenstein et al., 2021).

Smoking is a high-risk environmental factor for AMD. Dysregulation of VEGF and pigment epithelium-derived factor (PEDF) expression, two substances that promote and inhibit angiogenesis respectively, may lead to choroidal neovascularization and further development, causing vision loss. In a non-transformed human retinal pigment epithelium cell line, nicotine upregulated VEGF expression and downregulated PEDF expression through nAChRs. In the rat retinal pigment epithelium, nicotine upregulated the expression of VEGF and PEDF. By altering the ratio of the two growth factors, nicotine may play a role in the development of AMD (Pons and Marin-Castaño, 2011). Interestingly, nicotine treatment showed completely opposite results on VEGF expression in Klettner’s study, which may be related to different animal models. As shown in Figure 7D, perfused organ cultures of retina/retinal pigment epithelium/choroid treated with different concentrations of nicotine showed varying degrees of reduction in VEGF expression. Among them, 10 μM nicotine treatment had the most significant effect on inhibiting VEGF expression. Another study showed that on exposing mice to nicotine, VEGF, PDGF, or a combination of one of these factors with nicotine, nicotine increased choroidal neovascularization size and vascularity, especially in aged mice. This effect was blocked by hexamethonium, a non-specific nicotinic receptor antagonist. In addition, the growth of choroidal vascular SMCs was significantly increased after exposure to the combined treatment with PDGF and nicotine (Suñer et al., 2004). Taken together, nicotine may increase the size and severity of choroidal neovascularization in mouse models by enhancing the PDGF-mediated proliferation of choroidal SMCs.

#### 3.5.3 Diabetes

Diabetes is a chronic metabolic disease and one of the top ten causes of death in adults. Its development trend is getting more and more fierce and the number of cases worldwide has reached 425 million in 2017 (Saeedi et al., 2019). Diabetes includes three types, type 1 diabetes, type 2 diabetes, and gestational diabetes, of which type 2 diabetes accounts for more than 90% of all cases. The pathological features of type 2 diabetes are impaired insulin secretion, insulin resistance, or both (DeFronzo et al., 2015).

Epidemiological studies have shown that smokers are much more likely to develop diabetes than non-smokers. Studies have shown that nicotine-fed rats have increased circulating levels of glucagon and insulin, and are also accompanied by symptoms of glucose homeostasis disorders (Duncan et al., 2019). Among them, adrenaline plays a key role. By analyzing skeletal muscle biopsy samples from smokers and non-smokers, Bergman et al. found that smokers had increased Ser636 phosphorylation of IRS-1 and reduced PPAR-γ expression, resulting in reduced insulin sensitivity (Caligiuri and Kenny, 2021), which is consistent with the conclusion that nicotine aggravates insulin resistance in patients with type 2 diabetes and healthy smokers (Chen et al., 2023). In addition, patients with type 2 diabetes seem to metabolize nicotine faster, resulting in greater smoking and longer smoking time and further endangering human health (Keith et al., 2019).

Nicotine often enters the body through smoking; therefore, the parts that come into direct contact with tobacco smoke are often more prone to diseases such as periodontitis and lung cancer. In addition, nicotine has subtle effects on blood vessels, including damaging ECs, triggering inflammation, and eventually causing vascular obstruction. Excessive nicotine exposure from tobacco can increase the risk of preterm birth in pregnant women. Therefore, smokers may need to pay more attention to the health of relevant body parts and reduce tobacco intake appropriately. Table 1 lists the diseases associated with nicotine, experimental models used, nicotine doses, administration methods, influencing factors, and the effects of nicotine.

**TABLE 1:** Overview of the toxic effects of nicotine in different diseases.

Diseases	Model	Nicotine dosage	Administration	Factors	Effect	Ref.
Small cell lung cancer	Human SCLC cell lines (GLC8, NCI-N592 and NCI-H-69)	100 nM	*In vitro*	Serotonin	Stimulates serotonin release and SCLC proliferation	Codignola et al. (1994)
Non-small cell lung cancer	H157 and H1703 cells	100 nM	*In vitro*	Cyclin D1, PKB	Increases proliferation	Tsurutani et al. (2005)
Colon adenocarcinoma	HT-29 cells	10 nM, 100 nM,1,000 nM	*In vitro*	α7nAChR, TH, adrenaline	Stimulates proliferation and adrenaline production	Wong et al. (2007)
Gastric tumor	Athymic nude mice implanted with AGS	50 or 200 mg/mL in drinking water	Oral	ERK, COX-2, VEGF	Promotes gastric tumor growth and neovascularization	Shin et al. (2004)
Atherosclerosis	Primary human umbilical vein ECs and SMCs	1 μM	*In vitro*	α7nAChR, NF-κB	Induces SMC cytoskeleton protein up-expression	Wang et al. (2013)
Atherosclerosis	Human aortic endothelial cells	0.1 μM, 1 μM	*In vitro*	NLRP3, ASC, caspase-1, IL-1β, IL-18	Activates NLRP3-ASC inflammasome and pyroptosis	Wu et al. (2018)
Preterm birth	776,836 live singleton births in Sweden from 1999 to 2009	Snuff	Sniff	—	Increases the risk of preterm birth	Baba et al. (2012)
Sudden infant death syndrome	Adult female rats on gestation	2.1 mg/d	Osmotic minipump	Hypoxia/hypercapnia	Elicits an increase in excitatory neurotransmission to cardiac vagal neurons	Huang et al. (2005)
Chronic obstructive pulmonary disease	Human airway smooth muscle cells	0.1 mM, 1 mM, 5 mM, 10 mM, 50 mM	*In vitro*	α5nAChR, TRPC3	Promotes proliferation	Jiang et al. (2019)
Tuberculosis	Prenatal exposure	3 mg/kg	Administeration	α7nAChR, PI3K, PKB, β-catenin	Induces β-catenin level increase and thymopoiesis abnormalities	Wen et al. (2022)
Periodontitis	Human periodontal ligament cells	LPS (1 μg/mL) and nicotine (5 mM)	*In vitro*	HIF-2α, NO, PGE_2_, TRAP	Stimulates inflammatory response and osteoclastic differentiation	Bae et al. (2015)
Periodontitis	Human periodontal ligament cells	LPS (1 μg/mL) and nicotine (5 mM)	*In vitro*	PIN1, NO, PGE_2_, COX-2, NF-κB	Stimulates inflammatory response and osteoclastic differentiation	Cho et al. (2015)
Age-Related Macular Degeneration	C57BL/6 mice treated with laser	100 μg/mL in drinking water	Oral	PDGF, MMP2	Increases size and vascularity of choroidal neovascularization	Suñer et al. (2004)

Abbreviations: ERK, extracellular signal-regulated kinase; PI3K, phosphatidylinositol-3-kinase; NF-κB, nuclear factor kappa-B; VEGF, vascular endothelial growth factor; PKB, protein kinase B; TH, tyrosine hydroxylase; AP-1, activator protein-1; COX-2, cyclooxygenase-2; PGE2, prostaglandin E2; EC, endothelial cells; SMC, smooth muscle cells; LPS, lipopolysaccharide; PDGF, platelet-derived growth factor; ASC, apoptosis-associated speck-like protein containing CARD; HIF-2α, hypoxia-inducible factor-2α; PIN1, peptidyl-prolyl cis/trans isomerase NIMA-interacting protein 1; SCLC, small cell lung cancer; AGS, a poorly differentiated human gastric adeno-carcinoma cell line; NLRP3, NOD-like receptor thermal protein domain associated protein 3; TRPC3, transient receptor potential canonical 3; TRAP, tartrate-resistant acid phosphatase; MMP2, matrix metalloproteinase 2.

## 4 Pharmacodynamics: the beneficial effects of nicotine on the human body

**FIGURE 8:**
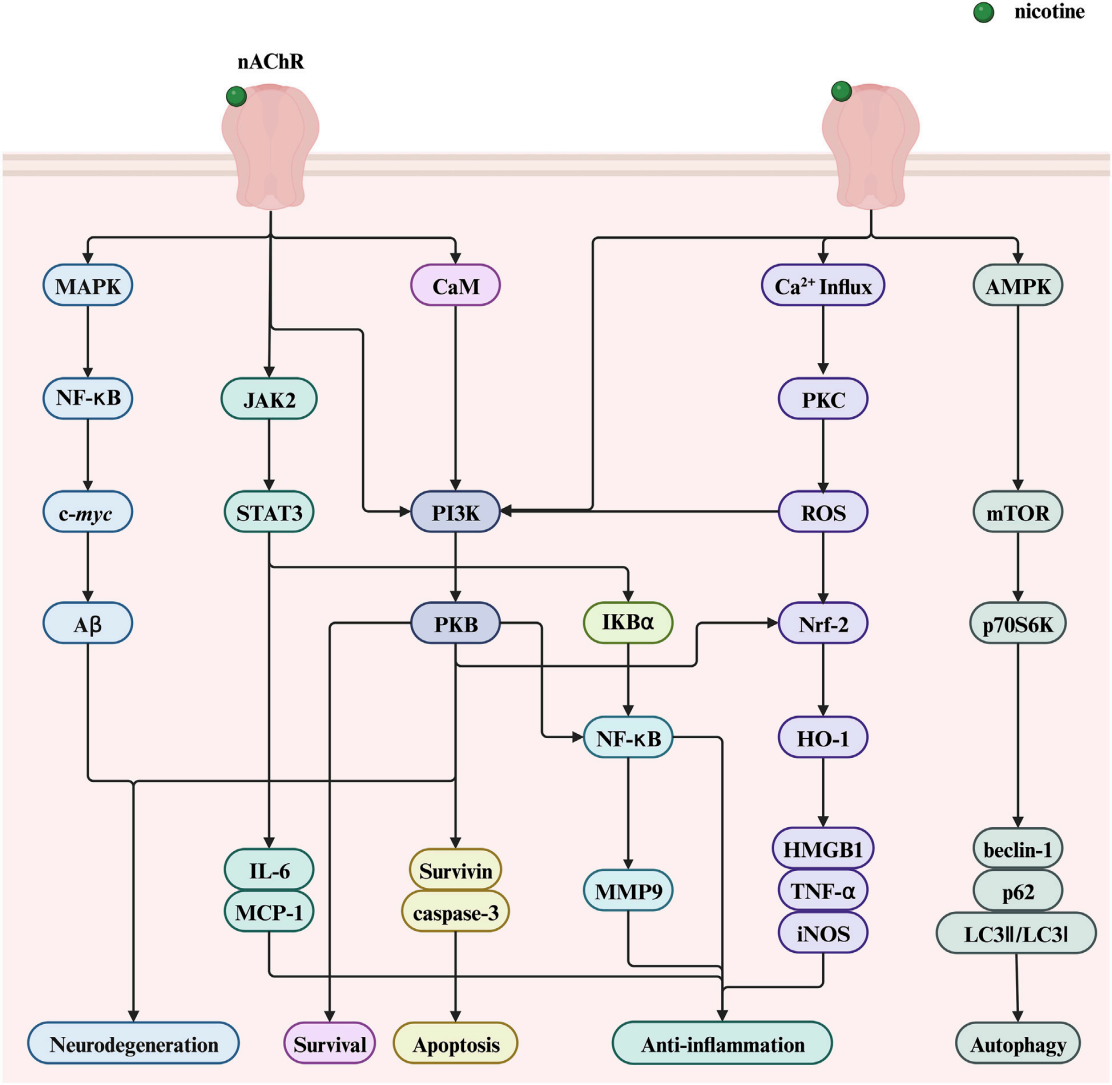
The therapeutic potential of nicotine and related signaling pathways. Created with BioRender.com. (Abbreviations: PI3K, phosphatidylinositol-3-kinase; NF-κB, nuclear factor kappa-B; PKB, serine/threonine kinase; STAT3, signal transducer and activator of transcription 3; ROS, reactive oxygen species; AMPK, AMP-activated protein kinase; JAK2, Janus kinase 2; CaM, calmodulin; IκBα, nuclear factor kappa B alpha; MCP-1, monocyte chemoattractant protein-1; HMGB1, high-mobility group box 1; HO-1, heme oxygenase-1; DSS, dextran sodium sulfate; nAChR, nicotinic acetylcholine receptor; MAPK, mitogen-activated protein kinase; NF-κB, nuclear factor kappa-B; Aβ, amyloid beta; IL-6, interleukin-6; MMP9, matrix metalloproteinase 9; PKC, protein kinase C; ROS, reactive oxygen species; Nrf2, nuclear factor erythroid 2-related Factor 2; TNF-α, tumor necrosis factor-alpha; mTOR, mechanistic target of rapamycin; p70S6K, p70 ribosomal protein S6 kinase).

Despite its adverse effects leading to the development of various diseases, nicotine exhibits potential therapeutic and pharmacological benefits in some diseases. This section highlights the therapeutic potential of nicotine, mainly focusing on two aspects: nervous and immune system diseases. Figure 8 illustrates the molecular mechanisms underlying the positive roles of nicotine in related diseases, including regulating various genes, transcription factors, and proteins. This figure helps readers more intuitively understand the therapeutic effect of nicotine in related diseases.

### 4.1 Nervous system diseases

**FIGURE 9:**
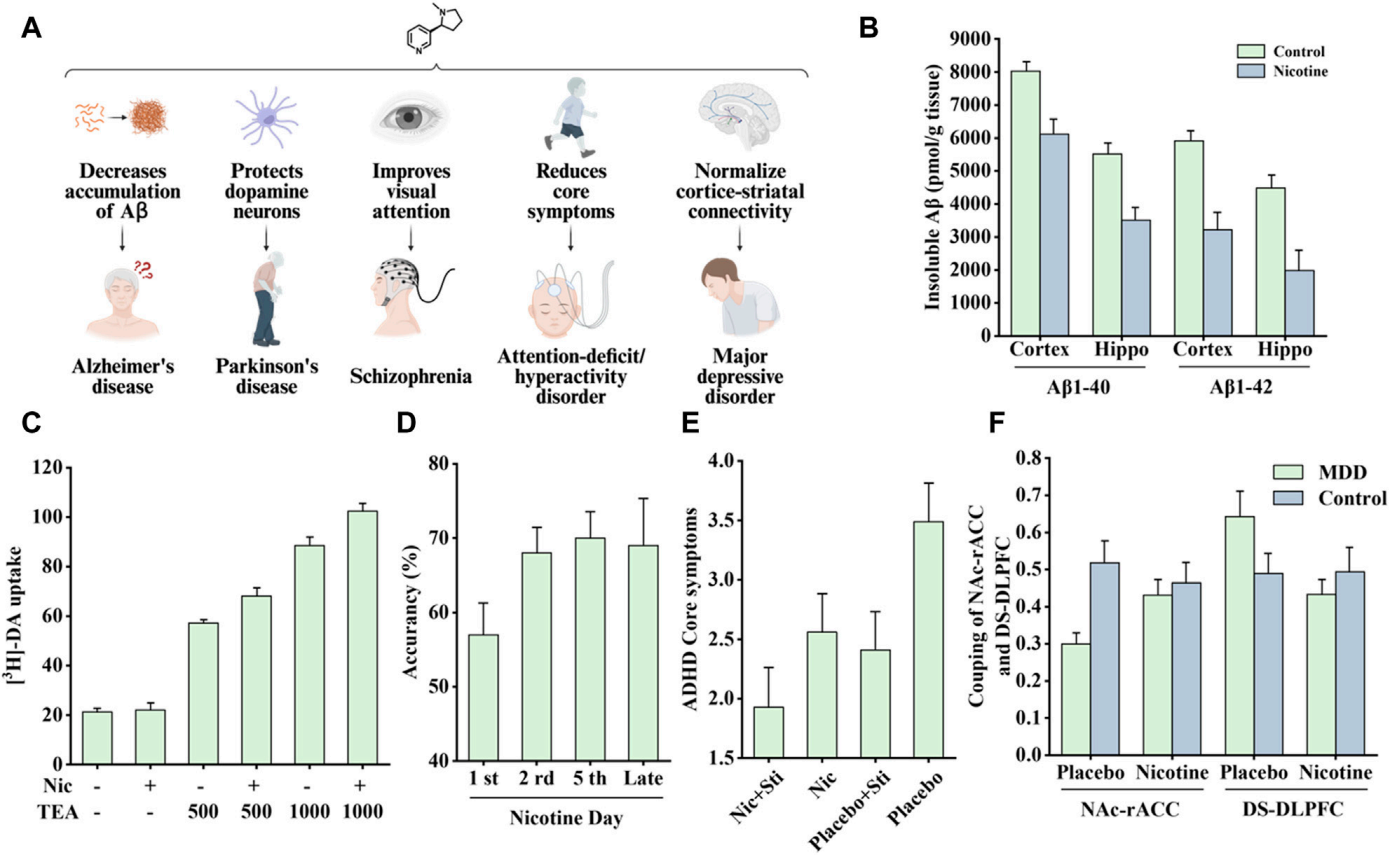
Effects of nicotine on nervous system diseases and related data graphs. **(A)** The effects of nicotine on the nervous system. Created with BioRender.com. **(B)** The level of insoluble amyloid beta (Aβ) in the cortex and hippocampus of transgenic mice under nicotine treatment. Reprinted (adapted) with permission from Ref. (Liu et al., 2007). Copyright 2007, Wiley-VCH. **(C)** Dopamine cell survival under nicotine and tetraethyl ammonium (TEA) treatment. Reprinted (adapted) with permission from Ref. (Toulorge et al., 2011). Copyright, 2011, Wiley-VCH. **(D)** Visual attention improvement in rats under nicotine treatment. Reprinted (adapted) with permission from Ref. (Bueno-Junior et al., 2017). Copyright, 2017, Springer Nature. **(E)** The average attention-deficit/hyperactivity disorder (ADHD) core symptoms under different treatments. Reprinted (adapted) with permission from Ref. (Gehricke et al., 2006). Copyright, 2006, Oxford University Press. **(F)** The coupling of nucleus accumbens-anterior cingulate cortex (NAc-rACC) and dorsal striatum-dorsolateral prefrontal cortex (DS-DLPFC) in the major depressive disorder (MDD) and healthy control groups under nicotine treatment and placebo. Reprinted (adapted) with permission from Ref. (Janes et al., 2018). Copyright, 2018, Springer Nature. (Abbreviations: Hippo, hippocampus; Nic, nicotine; DA, dopamine; Sti, stimulants).

Nicotine can extensively improve cognition, which has long attracted the interest of researchers. This section introduces the therapeutic effects of nicotine on Alzheimer’s disease (AD), Parkinson’s disease (PD), schizophrenia, attention-deficit/hyperactivity disorder (ADHD), and major depressive disorder (MDD). The associated therapeutic effects are shown in Figure 9A.

#### 4.1.1 Alzheimer’s disease

AD is a typical neurodegenerative disorder. In the early stages of the disease, it often manifests as learning and memory decline and mild language and movement impairments. As the disease progresses, patients eventually lose the ability to live independently and remain in bed for a long time until death. (Alan et al., 2023).

AD is marked by the deposition of extracellular amyloid beta (Aβ) and the aggregation of tubulin tau in neurons (Knopman et al., 2021). Among them, Aβ is produced in the cracking process of amyloid precursor protein (APP) and further accumulates to form amyloid fibers, causing neurotoxicity. In addition, α7nAChR has a strong affinity for Aβ, which can promote Aβ to enter cells through endocytosis. Lysis occurs when neurons are overburdened, and Aβ is released extracellularly, where further accumulation creates plaques (Ma and Qian, 2019).

Epidemiological studies have shown a significant negative correlation between smoking and AD incidence (Fratiglioni and Wang, 2000). In a 4-week transdermal nicotine treatment for patients with AD, nicotine significantly improved the patient’s attention performance (White and Levin, 1999). In another study of APP (V717I) transgenic mice, nicotine treatment reduced the accumulation of insoluble Aβ in the cortex and hippocampus, as shown in Figure 9B, which is associated with the MAPK/NF-κB/c-*myc* pathway mediated by α7nAChR. In addition, there appears to be a connection between cell cycle-related proteins and neuronal loss. Nicotine regulates the cell cycle and apoptotic processes to reduce neuronal loss by decreasing the mRNA and protein levels of cyclin D1 and CDK4 and the pro-apoptotic factors Bax and caspase-3 (Liu et al., 2007).

Another study published by Inestrosa et al. pointed out that nicotine prevents Aβ-induced neuronal synapse damage through the α7nAChR/PI3K pathway. In addition, Wnt/β-catenin signaling may be crucial in neuroprotection. The Wnt signaling pathway can regulate synaptic transmission and plasticity, and increased β-catenin expression can improve the structure of dendrites. Activating Wnt can promote α7nAChR expression, and nicotine prevents Aβ-induced β-catenin reduction through α7nAChR. In addition, nicotine has been shown to improve memory in APP/PS1 transgenic mice (Inestrosa et al., 2013; Inestrosa and Varela-Nallar, 2014).

#### 4.1.2 Parkinson’s disease

PD is the second most prevalent neurodegenerative disorder, affecting 2%–3% of people >65 years of age. Patients often present with motor symptoms such as bradykinesia, rigidity, resting tremor, and persistent cognitive, autonomic, and mood disturbances. Neuropathological features include striatal dopamine deficiency due to loss of substantia nigra dopaminergic neurons and extensive intracellular α-synuclein aggregates (Dickson et al., 2009). Neuronal damage is associated with multiple factors such as α-synuclein aggregates, mitochondrial dysfunction, and neuroinflammation (Poewe et al., 2017b).

Studies have shown a significant negative correlation between PD incidence and smoking, coffee consumption, and drinking alcohol (Nicoletti et al., 2010). Further research showed that nicotine can protect animal models of PD from nigrostriatal damage. In rats, administering nicotine before injury effectively reduced striatal dopamine loss (Costa et al., 2001), and in MPTP-treated monkeys, chronic oral nicotine decreased dopamine turnover, enhanced synaptic plasticity, and improved substantia nigra function after striatal injury (Quik et al., 2006), but this protective effect only occurred before striatal injury. This indicated that nicotine can only exert a protective effect on striatal injury rather than a repairing effect (Huang et al., 2009).

Nicotine-mediated neuroprotection also involves nAChRs and is subsequently divided into calcium-dependent and calcium-independent pathways (Quik et al., 2012). The calcium-dependent pathway involves an increase in intracellular Ca^2+^ concentration, which may be mediated by nAChR or other membrane channels, thereby activating different downstream signaling channels to exert neuroprotective effects. In a study by Toulorge et al., nicotine promoted cell survival by activating downstream calmodulin (CaM)/PI3K/PKB-dependent signaling. As shown in Figure 9C, in the presence of K^+^-channel blocker tetraethyl ammonium (TEA), nicotine treatment exerted more significant neuronal protective effects (Toulorge et al., 2011). The calcium-independent pathways involve the JAK2-STAT3 signaling cascade. Blocking downstream inhibitor of nuclear factor kappa B alpha (IκBα) phosphorylation and NF-κB translocation can reduce neuronal inflammation, which depends on α4β2nAChR (Hosur and Loring, 2011).

As reported by Holmes et al., nicotine selectively affected the non-smokers’ controlled semantic processing that is impaired in PD patients in a cognitive test, which may be attributed to nicotine’s enhancement of expectation or inhibition mechanism (Holmes et al., 2011). Villafane et al. found that chronic high-dose transdermal nicotine treatment in five PD patients resulted in improved motor scores and reduced dopaminergic responses, but with side effects such as nausea and vomiting (Villafane et al., 2007). Itti et al. studied six PD patients who received nicotine therapy for 1 year and found that motor function improved at 3 months, but the improvement was less sustained after 1 year. Dopamine transporter imaging showed that the density of striatal neurons in the treated patients was relatively stable, indicating that nicotine has pharmacological and neuroprotective effects (Itti et al., 2009). PD patients often have symptoms of low blood pressure. DiFrancisco et al. found that patients’ systolic blood pressure would increase within 10 min after taking 4 mg nicotine gum and remain elevated within 90 min by studying 10 subjects (DiFrancisco-Donoghue et al., 2019). Another study on two PD patients found that nicotine gum and patch treatment improved the patients’ motor delay and confusion (Fagerström et al., 1994). Nicotine gum is rapidly absorbed and is expected to become a new type of treatment. Although nicotine has shown some value in the treatment of PD, it seems that larger-scale population trials are needed to confirm its application if to be widely promoted.

#### 4.1.3 Schizophrenia

Schizophrenia is a serious mental illness classified into three categories: positive, negative, and cognitive. Positive symptoms are often accompanied by delusions, hallucinations, and behavioral disturbances; negative symptoms are often accompanied by depression, anhedonia, and social withdrawal; and cognitive symptoms mainly manifest as cognitive dysfunction (Kahn et al., 2015). Presently, the etiology of the disease remains unclear, and patients mainly rely on drug therapy. Drug therapy can improve positive symptoms but cannot effectively relieve negative and cognitive symptoms (Stępnicki et al., 2018).

Smoking is a high-risk factor for patients with schizophrenia; however, the biological mechanisms underlying this association remain unclear. Liu et al. investigated the relationship between alterations in intrinsic brain activity associated with schizophrenia pathology and nicotine addiction. The authors found that smoking reversed intrinsic brain activity in the right striatum and prefrontal cortex, consistent with the pharmacological theory of schizophrenia. Furthermore, addictive effects are independent of the disease (Liu et al., 2018). Interestingly, Whitton et al. found that dopamine D2 receptor antagonists reduced the reward-enhancing effects of nicotine and were associated with increased smoking rates in patients, which explains why patients taking potent dopamine D2 receptor antagonists often exhibit more symptoms of nicotine dependence (Whitton et al., 2019).

Smucny et al. examined the effect of nicotine on the connectivity within a ventral attention network. When patients performed a selective attention task, the connectivity between the ventral parietal cortex seeds and the inferior frontal gyrus decreased, and nicotine increased this connectivity (Smucny et al., 2016). Another study found that in a mouse model, nicotine treatment increased gamma oscillations in the prefrontal cortex, which was associated with enhanced visual attention. As shown in Figure 9D, nicotine exposure increased visual attention accuracy in rats (Bueno-Junior et al., 2017).

The prefrontal cortex is fundamental to higher cognitive processes and regulated by nAChRs. A genome-wide association study found that single-nucleotide polymorphisms (SNPs) in the CHRNA5 gene encoding the α5nAChR subunit were associated with an increased risk of smoking and schizophrenia. In mice expressing α5-SNP and α5-knockout mice, interneuron inhibition of layer II/III pyramidal neurons increases, activity reduces, and frontal function declines. However, chronic nicotine administration modulates layer II/III inhibition circuitry through nAChR, reversing the above effects (Koukouli et al., 2017). A subsequent study showed that these prefrontal cortex circuit dynamics involved changes in the structure of active-state stability and that changes in amplitude were associated with reduced cone firing rates in α5 SNP mice. In addition, this experiment demonstrated that nicotine induced the desensitization and upregulation of β2nAChR on somatostatin interneurons but not the activation of α5nAChR on vasoactive intestinal polypeptide interneurons, which explains why the activity of α5 SNP mice normalized after nicotine treatment. In addition, the study showed that nicotine withdrawal may exacerbate SNP-induced frontal hypocreasia (Rooy et al., 2021).

#### 4.1.4 Attention-deficit/hyperactivity disorder

ADHD is a common neurodevelopmental disorder caused by multiple genetic and environmental factors. Typical features include inattention and hyperactive impulsiveness, which affect approximately 5% of children and adolescents worldwide, with huge financial costs and family stress. For now, medication remains an effective way to reduce ADHD symptoms (Faraone et al., 2015).

Molecular genetic studies have shown that susceptibility to ADHD is associated with three genes: D4 dopamine receptor, D2 dopamine receptor, and dopamine transporter genes. Neurological deficits in children include executive function and working memory deficits, which may be associated with dysfunction in the frontal lobar cortex (Faraone and Biederman, 1998).

Nicotine has been shown to improve attentional performance in this disorder. In a rat model, nicotine administration improved working memory in the radial-brachial maze, an effect associated with α4β2nAChR and α7nAChR in the ventral hippocampus and basolateral amygdala. Local infusion of α4β2nAChR and α7nAChR antagonists induced working memory deficits, whereas ventral hippocampal α4β2nAChR blockade-induced working memory deficits were reversed by systemic nicotine treatment (Levin, 2002). In addition, patients with ADHD appear to have a higher risk of smoking and failure to quit, perhaps because of the “self-medication hypothesis.” Accordingly, patients’ active or continued exposure to cigarettes is attributed to nicotine in tobacco products that can supplement the lack of dopamine in the cortico-striatal pathway, thereby relieving symptoms. In addition, the nicotine analogs varenicline and bupropion improved ADHD-related symptoms and also supported the above hypothesis (Taylor et al., 2022). Nicotine patches and stimulant medications, alone or in combination, have been found to reduce concentration difficulties and core symptoms in patients with ADHD (Figure 9E) (Gehricke et al., 2006). Another acute nicotine treatment in patients with ADHD revealed that these patients had improved recognition memory, increased delay tolerance, and a corresponding reduction in reaction time in a stop-signal task (Potter and Newhouse, 2008). A recent study pointed out that nicotine improved two pathways involving the VTA; one normalized abnormal activity in animal models of ADHD, and the other induced atypical brain responses in animals with ADHD (Poirier et al., 2017a).

#### 4.1.5 Major depressive disorder

MDD is a mental illness often accompanied by depression, loss of interest, cognitive impairment, fatigue, difficulty in sleeping, loss of appetite, and other symptoms. The incidence rate in women is generally higher than that in men, and genetic factors and childhood abuse can increase the incidence rate (Seedat et al., 2009; Li et al., 2016). The disease is associated with altered brain volume in the hippocampus, altered function of the cognitive control and affective-salience networks, and disturbances of the hypothalamic-pituitary-adrenal axis and immune system (Otte et al., 2016).

MDD and nicotine dependence are highly comorbid, and their causal link remains unclear. Markou et al. believe that it may be associated with changes in the function of neurotransmitters in limbic brain structures (Markou et al., 1998). Cardenas et al. further speculated that the two may be associated with the dysfunctional dopaminergic brain reward system. However, nicotine treatment did not change the brain’s stimulus response to d-amphetamine, and the severity of depression was highly correlated with this reward effect (Cardenas et al., 2002). A clinical and preclinical study on nicotine and depression showed that nicotine may share some of the properties of antidepressants, whereas some antidepressants are also effective smoking cessation agents; therefore, MDD and nicotine dependence may share some related neuronal substrates (Laje et al., 2001; Dome et al., 2010). In a recent study, nicotine reportedly improved neurobiological dysfunction in the cortico-striatal circuit associated with MDD. Specifically, in patients with MDD, connectivity between the nucleus accumbens (NAc) and the rostral anterior cingulate cortex (rACC) is reduced, whereas connectivity between the dorsal striatum (DS) and dorsolateral prefrontal cortex (DLPFC) is increased. Acute nicotine treatment normalized these pathways to the levels observed in healthy controls (Figure 9F).

Notably, the effect of nicotine on NAc-rACC connectivity was associated with anhedonia, a network implicated in rewarding effects (Janes et al., 2018). An electroencephalogram study involving patients with MDD showed decreased activation of the left and right frontal cortices, in which the left frontal cortex was associated with positive emotion regulation. Nicotine treatment normalized these changes, including a modest increase in right hemisphere alpha1 amplitude and reduced left-biased alpha1 amplitude asymmetry (Jaworska et al., 2011).

### 4.2 Immune system diseases

**FIGURE 10:**
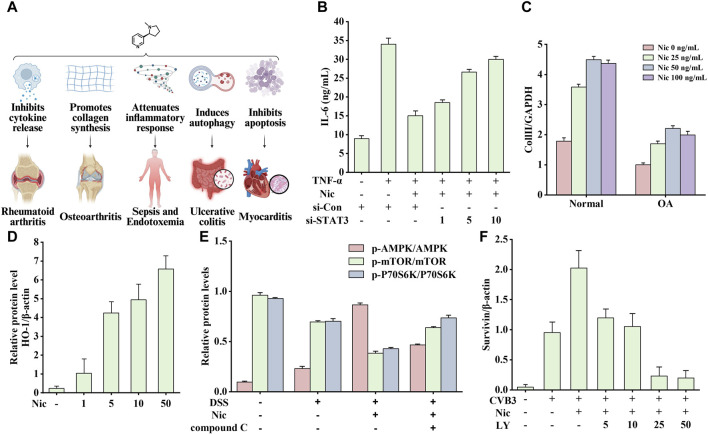
Effects of nicotine on immune system diseases and related data graphs. **(A)** The effects of nicotine on immune system diseases. Created with BioRender.com. **(B)** Interleukin-6 (IL-6) protein concentration in culture supernatants under different treatments. Reprinted (adapted) with permission from Ref. (Li et al., 2015). Copyright 2015, Springer Nature. **(C)** The expressed mRNA of type II collagen (Coll II in cells under nicotine treatment. Reprinted (adapted) with permission from Ref. (Ying et al., 2012a). Copyright 2012, Springer Nature. **(D)** The expression of heme oxygenase-1 (HO-1) in macrophages under nicotine treatment. Reprinted (adapted) with permission from Ref. (Tsoyi et al., 2011). Copyright 2011, Mary Ann Liebert, Inc. **(E)** The relative protein levels of p-AMP-activated protein kinase (AMPK), p-mechanistic target of rapamycin (mTOR), and p-p70 ribosomal protein S6 kinase (p70S6K) in cells under dextran sodium sulfate (DSS), nicotine, and compound C treatment. Reprinted (adapted) with permission from Ref. (Gao et al., 2020). Copyright 2020, Elsevier B.V. **(F)** Survivin expression in cells under nicotine, coxsackievirus B3 (CVB3), and LY294002 treatment. Reprinted (adapted) with permission from Ref. (Li P. et al., 2019). Copyright 2019, Springer Nature. (Abbreviations: TNF-α, tumor necrosis factor-alpha; Nic, nicotine; si-Con, Control siRNA; si-STAT3, STAT3 siRNA; OA, osteoarthritis; LY, LY294002).

Inflammation involves multiple genes and signaling pathways. In addition, it promotes the occurrence and development of various diseases to varying degrees. Nicotine plays an active role in various immune disorders because of its broad anti-inflammatory properties. This section describes the therapeutic effects of nicotine in rheumatoid arthritis (RA), OA, sepsis, endotoxemia, ulcerative colitis (UC), and myocarditis. The related effects are shown in Figure 10A, which may be associated with the different models, concentrations, durations, and corresponding tissues and organs.

#### 4.2.1 Arthritis

Arthritis refers to any condition affecting the joints, including joint pain and stiffness, often accompanied by redness, warmth, and swelling. RA and OA are two typical joint diseases (March et al., 2014).

RA is a chronic inflammatory disease characterized by synovial inflammation, cartilage destruction, and bone erosion. It is an autoimmune disease; when the peptide undergoes a post-translational modification process of citrullination, the presentation of the newly generated peptide by the antigen-presenting cell activates the immune system and induces the production of autoantibodies, mainly immunoglobulin G (rheumatoid factor and anti-citrullinated protein antibodies). Subsequently, fibroblast-like synoviocytes (FLSs) and antigen-presenting cells are activated to produce inflammatory factors, leading to synovial inflammation, whereas a sustained immune response eventually leads to cartilage degeneration and bone erosion (Schellekens et al., 1998; Smolen et al., 2018).

The most typically used animal model of RA is the collagen-treated DBA/1 mouse, which often exhibits cartilage damage, cell infiltration, and bone destruction similar to human RA symptoms. Li’s study showed that nicotine inhibited the secretion of IL-6 and monocyte chemoattractant protein-1 (MCP-1) in RA-FLSs through the α7nAChR/JAK2/STAT3 pathway to exert anti-inflammatory effects (Li et al., 2015). The pro-inflammatory cytokine IL-6 regulates T lymphocytes to produce osteoclast factors and inflammation-induced bone marrow osteoclast differentiation (Wong et al., 2006). However, the chemokine MCP-1 regulates the RA process by recruiting monocytes (Ogata et al., 1997). As shown in Figure 10B, STAT3-specific small interfering RNA transfection (STAT3-siRNA) leads to a significant decrease in STAT3 expression, while leading to an overall anti-inflammatory degree of nicotine (inhibition of TNF-α-induced IL-6 increase) reduction compared with that in the control group (Con-siRNA). The higher the dose, the weaker the anti-inflammatory effect of nicotine. Therefore, the anti-inflammatory effects of nicotine require STAT3 activation.

Cholinergic anti-inflammatory pathways play a crucial protective role in disease development. The vagus nerve’s efferent activity promotes the release of acetylcholine from various organs, which inhibits the release of pro-inflammatory cytokines by binding to receptors on the surface of macrophages (Tracey, 2002). Similar to acetylcholine, nicotine can exert anti-inflammatory effects by inhibiting the release of inflammatory factors through the vagus nerve. In Maanen’s study, nicotine (400 mg/kg, intraperitoneal injection) administration for 7 days reduced bone degradation and TNF-α expression in the synovial tissue, and this process may be associated with the specific effect of the vagus nerve on α7nAChR, because cutting the vagus nerve led to the deteriorating condition of mice (van Maanen et al., 2009). In another experiment, nicotine (0.1, 1, and 10 μM) decreased the expression of IL-6 and IL-8 in TNF-α-induced FLSs and inhibited the translocation of NF-κB from the cytoplasm to nucleus (Zhou et al., 2012). In addition, the combined treatment with thymol (50 mg/kg) and nicotine (1.25 mg/kg) showed a better therapeutic effect and led to a decreased expression of rheumatoid factor, myeloperoxidase, and IL-1 (Golbahari and Abtahi Froushani, 2019).

Multiple risk factors are responsible for OA development, and its prevalence increases sharply with age, with a higher incidence in women. OA affects the entire joint, including articular cartilage, subchondral bone, and synovium. Chondrocytes cause changes in the cartilage matrix. When chondrocytes cannot repair the damaged cartilage matrix, they secrete matrix-degrading enzymes, ROS, cytokines, and chemokines, which further trigger synovial inflammation and lead to cartilage degeneration (Martel-Pelletier et al., 2016).

Nicotine tends to positively affect OA, in which α7nAChR plays a key role. In a rat model of early OA, nicotine (50 mg/mL in drinking water) promoted matrix production, ameliorated cartilage destruction, decreased TNF-α levels in the serum and synovial tissue, and upregulated the expression of α7nAChR in synovial tissue, showing promising therapeutic potential (Gu et al., 2015). In the same year, Liu et al. conducted a more in-depth exploration and found that nicotine (10 μM) significantly attenuated joint degeneration and p38, Erk1/2, and JNK MAPK activation of chondrocytes in monosodium iodoacetate-treated mice, all of which were reversed by α7nAChR antagonist methyllycaconitine (Liu et al., 2015). Elevated MMP9 expression is a sign of OA joint inflammation. Nicotine inhibits LPS-induced NF-κB translocation through the α7nAChR/PI3K/PKB pathway, thereby reducing MMP9 gene expression. In addition, mice cartilage degeneration and mechanical allodynia were reduced (Teng et al., 2019).

The imbalance between autophagy and apoptosis in chondrocytes may accelerate cartilage degeneration, and the expression of α7nAChR was found to be downregulated in the knee articular cartilage tissue of patients, leading to mTOR phosphorylation and increased possibility of apoptosis. Nicotine-activated α7nAChRs/mTOR signaling pathway alleviates pain and cartilage degeneration and regulates the balance between apoptosis and autophagy (Liu et al., 2021). Furthermore, as shown in Figure 10C, nicotine promoted type II collagen expression in chondrocytes isolated from normal humans and patients with OA in a dose-dependent manner. OA chondrocytes were expressed less than normal cells (Ying et al., 2012a). The authors also found that appropriate nicotine concentrations enhanced the cartilage differentiation ability of bone marrow stromal stem cells (Ying et al., 2012b).

#### 4.2.2 Sepsis and endotoxemia

Sepsis typically results from a dysregulated systemic inflammatory and immune response to infection, ultimately leading to organ damage. The exact process is not fully understood but mainly includes two phases: inflammatory outbreaks and immunosuppression. An inflammatory outbreak occurs mainly when infection-derived microorganisms are recognized, simultaneously triggering multiple signaling cascades to release multiple inflammatory cytokines, leading to vasodilation, tissue damage, and multiorgan failure. In the next stage, patients show chronic suppression of the innate and adaptive immune systems, resulting in severe leukocyte apoptosis (Hotchkiss et al., 2016).

In high-income countries, 31.5 million cases and 5.3 million deaths are reported annually (Hotchkiss et al., 2016). In multiple sepsis and experimental models, nicotine improved survival and attenuated multiple organ dysfunction under different conditions. This suggests that nicotine could potentially be developed as a drug for treating sepsis.

Cholinergic pathways are involved in nicotine-mediated protection. The vagus nerve is critical in regulating innate immune responses to bacterial infections. Septic peritonitis was induced in mice by intraperitoneal injection of live *Escherichia coli*, and the subsequent effects were observed by transection of the vagus nerve or nicotine treatment. Nicotine preconditioning reduces cytokine release during septic peritonitis, independent of vagal integrity. Further research showed that neutrophil influx, pro-inflammatory cytokine levels, and liver damage increased after vagus nerve-cutting treatment, whereas nicotine preconditioning reversed these effects (van Westerloo et al., 2005).

The toll-like receptor (TLR) is involved in initiating inflammatory responses. In a mouse model of sepsis established by cecal ligation and puncture, intraperitoneal injection of nicotine attenuated the increase in TLR4 protein and gene expression. In addition, the regulation of TLR4 gene expression in macrophages determines the strength and timing of the response to endotoxins, among which the myeloid-specific factor PU.1 serves as an important transcription factor. Nicotine inhibited PU.1-binding activity in macrophages via α7nAChR, and PU.1 inhibition was observed at 6 h after cecal ligation and puncture to coincide with a reduced TLR4 mRNA expression (Pedchenko et al., 2005). Based on the above results, it can be reasonably speculated that nicotine-regulated TLR4 transcription is mediated by PU.1.

High-mobility group box 1 (HMGB1) is a lethal cytokine in the cecal ligation and puncture-induced sepsis model. In Konstantin’s study, nicotine was found to exert anti-inflammatory effects through the α7nAChR/Ca2+ influx/PKC/ROS/PI3K/PKB/nuclear factor erythroid 2-related Factor 2 (Nrf2) pathway by inducing macrophages to produce heme oxygenase-1 (HO-1), thereby decreasing the release of HMGB1, TNF-α, and iNOS, which ultimately increases survival in mice with sepsis. As shown in Figure 10D, nicotine dose-dependently increased the relative protein level of HO-1 (Kim et al., 2014). In another experiment, nicotine inhibited the release of HMGB1 from macrophages induced by endotoxin or TNF-α by activating cholinergic signaling pathways, prevented NF-κB pathway activation, and improved the survival rate in experimental models of sepsis. A positive treatment effect can still be observed after disease onset. In addition, nicotine (400 μg/kg, intraperitoneal injection) alleviates clinical manifestations such as lethargy, diarrhea, piloerection, and curling up and prevents hypothermia and decreased hematocrit caused by endotoxemia (Wang H. et al., 2004).

Nicotine has also been shown to be beneficial in treating endotoxemia. Wistar rats were injected with LPS (5 mg/kg body weight) to establish an animal model of endotoxemia; the expression of TNF-α and IL-6 increased, and nicotine treatment could significantly inhibit these increased expressions. In addition, nicotine treatment (0.1 mg/kg) inhibited the increase in plasma alanine aminotransferase levels and restored diamine oxidase activity. Among them, α7nAChR was also involved in these anti-inflammatory and protective effects (Zhou et al., 2011).

Overall, nicotine appeared to improve sepsis and endotoxemia. As two fatal diseases, nicotine can be considered for emergency patients requiring life-saving care in the absence of specific drugs. However, the side effects of nicotine on the human body should be minimized.

#### 4.2.3 Ulcerative colitis

Recently, the annual incidence of UC has increased. UC is a typical chronic inflammatory bowel disease that extends from the rectum to the distal colon, where a long-term and persistent inflammatory response in the innermost intestinal mucosa develops, eventually leading to ulcers and bloody diarrhea (Kobayashi et al., 2020).

In patients with UC, the diversity of the microbiota and thickness of the mucus layer are reduced, the synthesis of tight junction proteins and the pore-forming protein claudin two is reduced, the barrier is impaired, and further development can lead to barrier breakdown. Subsequently, many microbes cross the epithelial barrier, activate macrophages and antigen-presenting cells, and attract neutrophils. Activated neutrophils form neutrophil extracellular traps that cause mucosal damage. Monocytes bind to and infiltrate the adhesion molecules expressed by the vascular endothelium and mature into macrophages. Macrophages secrete various cytokines, such as TNF, IL-12, and IL-6, which polarize T helper cells and promote further UC development (Kobayashi et al., 2020).

Generally, nicotine is beneficial for treating UC. In particular, transdermal nicotine patches or nicotine enemas used as therapeutic agents in patients have been shown to improve colitis histology and global clinical scores (Abdrakhmanova et al., 2010). Among these, 6 mg of oral and transdermal nicotine is well tolerated and represents the highest therapeutic dose used in clinical practice, with a low risk of adverse reactions in humans (Ingram et al., 2004).

The most typically used animal model of UC is the dextran sodium sulfate (DSS)-induced mouse or rat model, often accompanied by symptoms such as rectal bleeding, diarrhea, and weight loss. In C57BL/6J mice treated with 3% DSS, nicotine (0.1 mg/mL) administration through drinking water attenuated DSS-induced increases in mucosal vascular addressin cell adhesion molecule-1 (MAdCAM-1), vascular cell adhesion molecule 1, and leukocyte recruitment, and disease activity index and histological scores were also attenuated. Recruiting leukocytes to the inflamed colon is essential to UC disease progression. These results may suggest that nicotine treatment can improve colitis by inhibiting MAdCAM-1 expression on inflamed colonic microvessels (Maruta et al., 2018). In another study, nicotine (10 μg/kg/day, gavage) modulated autophagy through the AMPK-mTOR-P70S6K signaling pathway to enhance the expression of LC3II/LC3I and beclin-1 and decrease the p62 protein level, which also reduced the disease activity score, body weight, histological damage score, and colon levels of inflammatory factors.

As shown in Figure 10E, nicotine treatment increased p-AMPK levels and decreased p-mTOR and p-P70S6K levels, and this effect was attenuated by compound C (AMPK inhibitor). Therefore, drugs targeting AMPK are expected to help treat UC. In addition, nicotine increased the expression of LC3II/LC3I and beclin-1 but decreased p62 protein levels, all associated with nicotine-promoted autophagy (Gao et al., 2020). In BALB/C mice, nicotine reduced the number and size of the colonic tumors associated with chronic colitis through the IL-6/Stat3/miR-21 signaling pathway. In addition, nicotine also attenuated colonic severity of inflammation (Hayashi et al., 2014).

Early reports mentioned that patients with UC had worsening disease after quitting smoking and improved after resuming smoking (Lakhan and Kirchgessner, 2011). In clinical practice, patients with moderate UC achieved relief after taking nicotine gum (Wolf and Lashner, 2002), and transdermal nicotine-assisted methotrexate enema therapy was significantly superior to combined oral and rectal methotrexate therapy (Guslandi et al., 2002). Although transdermal nicotine shows potential for the treatment of UC, it is often associated with side effects such as nausea, dizziness, and headache. While added to conventional treatment as an adjunctive treatment, nicotine enemas have shown almost no side effects (Green et al., 1997).

#### 4.2.4 Myocarditis

Myocarditis may be accompanied by various symptoms, ranging from dyspnea or chest pain to severe cardiogenic shock or death and dilated cardiomyopathy with sequelae of chronic heart failure. Myocarditis is usually caused by common viral infections; in specific cases, it can also be caused by other pathogens, toxic or hypersensitive drug reactions, or sarcoidosis. Currently, specific treatments for myocarditis are lacking (Cooper, 2009).

The cholinergic anti-inflammatory pathway effectively protects the myocardium from viral infections. When pathogens invade the body, damaged tissues produce and release inflammatory factors that act on the afferent sensory nerve of the solitary nucleus, and the efferent vagus nerve is activated to release acetylcholine. Subsequently, acetylcholine binds to α7nAChR on the surface of inflammatory cells, inhibits the synthesis and release of pro-inflammatory factors, reduces inflammatory responses, and prevents tissue damage and death (Oke and Tracey, 2009).

In a coxsackievirus B3 (CVB3) murine myocarditis model, nicotine activated α7nAChR, increased STAT3 phosphorylation, reduced the expression of TNF-α and IL-6, and attenuated the damage due to viral myocarditis. Conversely, methyllycaconitine exerted an effect exactly opposite to that of nicotine (Cheng et al., 2014). To further study the dose-related effects of nicotine in mice with viral myocarditis, Li et al. administered 0.1, 0.2, or 0.4 mg/kg thrice daily for seven or 14 consecutive days and found that the mice survived a dose-dependent increase in TNF-α, IL-1β, IL-6, and IL-17A mRNA expression and protein levels, decreased myocardial inflammation, and improved left ventricular function (Li-Sha et al., 2015).

Interestingly, these nicotine-mediated changes were independent of vagal integrity. Right cervical vagotomy was shown to inhibit the cholinergic anti-inflammatory pathway, aggravate cardiomyopathy, and impair left ventricular function, and activation of the cholinergic pathway by nicotine treatment could reverse these changes; this process was dependent on α7nAChR (Li-Sha et al., 2017). Furthermore, cardiomyocyte apoptosis is critical for the development of CVB3-induced myocarditis. In another study, nicotine inhibited apoptosis by mediating the anti-apoptotic protein survivin and caspase-3 through the α3β4nAChR/PI3K/PKB pathway. As shown in Figure 10F, adding the PI3K inhibitor LY294002 decreased survivin levels, which worsened ventricular systolic function and reduced survival in mice compared with that in the nicotine control group. In addition, nicotinic agonists reduced CVB3 replication in a dose-dependent manner *in vitro*, suggesting that nAChRs may mediate a protective mechanism in myocarditis (Li P. et al., 2019).

Nicotine has been associated with positive effects on cognition and inflammation, which may benefit individuals with neurological and immune system disorders. As a stimulant, nicotine can bind to the acetylcholine receptors on neurons to promote the release of dopamine and alleviate various neurological diseases. Anti-inflammatory effects against some diseases asre associated with the cholinergic anti-inflammatory pathway. Nicotine reduces the release of various inflammatory cytokines by binding to the macrophage surface receptors. Table 2 summarizes the positive effects of nicotine on the abovementioned diseases, its dosage, and administration.

**TABLE 2:** The overview of nicotine’s therapeutic potential in different diseases.

Diseases	Model	Nicotine dosage	Administration	Factors	Effect	Ref.
Alzheimer’s disease	APP (V717I) transgenic mice	200 μg/mL in drinking water	Oral	MAPK, NF-κB, c-*myc*, α7nAChR	Decreases accumulation of β-amyloid	Liu et al. (2007)
Parkinson’s disease	Rat midbrain cultures	10 μM	*In vitro*	α7nAChR, cytosolic Ca^2+^	Affords neuroprotection to dopamine neurons	Toulorge et al. (2011)
Attention deficit hyperactivity disorder	ADHD combined type adults	7 mg nicotine patch	Affixes to the skin	—	Improves the Stop Signal Reaction Time measure	Potter and Newhouse (2008)
Major depressive disorder	Non-smokers with and without MDD	2 mg nicotine lozenge	Dissolves in the mouth	Normalizes NAc-rACC and DS–DLPFC connectivity	Normalizes cortico-striatal connectivity	Janes et al. (2018)
Rheumatoid arthritis	TNF-α stimulated RA-FLSs	10 μM	*In vitro*	JAK2, STAT3	Downregulates production of IL-6 and MCP-1	Li et al. (2015)
Rheumatoid arthritis	Mice with collagen-induced arthritis	50 μg/mL in drinking water	Oral	α7nAChR, TNF-α	Inhibits bone degradation and reduces TNF-α expression	van Maanen et al. (2009)
Osteoarthritis	C57BL/6J mice treated with MIA	0.5 or 1 mg/kg	Intraperitoneal injection	α7nAChR-PI3K-PKB-NF-κB-MMP9	Suppress MIA-induced cartilage degradation	Teng et al. (2019)
Osteoarthritis	Male Sprague-Dawley rats treated with MIA	1 mg/kg	Intraperitoneal injection	p38-ERK-JNK, α7nAChR	Alleviates MIA-induced joint degradation	Liu et al. (2015)
Ulcerative colitis	C57BL/6J mice treated with 3% DSS	100 μg/mL in drinking water	Oral	MAdCAM-1	Attenuate leukocyte recruitment	Maruta et al. (2018)
Ulcerative colitis	C57BL/6 mice treated with 3% DSS	10 μg/kg per day	Gavage	AMPK-mTOR-p70S6K	Regulates autophagy and improves colitis	Gao et al. (2020)
Sepsis and Endotoxemia	Mice treated with cecal ligation and puncture	400 μg/kg	Intraperitoneal injection	α7nAChR-PI3K-PKB	Attenuates organ failure and suppresses inflammatory cytokines	Kim et al. (2014)
Sepsis and Endotoxemia	RAW 264.7 cells	10 µM	*In vitro*	PI3K, PKB, Nrf2	Upregulates HO-1 and provides anti-inflammatory action	Tsoyi et al. (2011)
Myocarditis	CVB3-infected neonatal rat cardiomyocytes	1 μM	*In vitro*	α3β4nAChR, PI3K, PKB	Protects cardiomyocytes from CVB3-induced apoptosis	Li et al. (2019b)

Abbreviations: MAPK, mitogen-activated protein kinase; PI3K, phosphatidylinositol-3-kinase; NF-κB, nuclear factor kappa-B; PKB, protein kinase B; STAT3, signal transducer and activator of transcription 3; AMPK, AMP-activated protein kinase; JNK, c-Jun N-terminal kinase; JAK2, Janus kinase 2; ADHD, attention-deficit/hyperactivity disorder; MDD, major depressive disorder; APP, amyloid precursor protein; RA, rheumatoid arthritis; FLS, fibroblast-like synoviocytes; MCP-1, monocyte chemoattractant protein-1; heme oxygenase-1; DSS, dextran sodium sulfate; MAdCAM-1, mucosal vascular addressin cell adhesion molecule-1; CVB3, coxsackievirus B3; NAc, nucleus accumbens; rACC, rostral anterior cingulate cortex; DS, dorsal striatum; DLPFC, dorsolateral prefrontal cortex; TNF-α, tumor necrosis factor-alpha; IL-6, interleukin-6; MIA, monosodium iodoacetate; α7nAChR, α7 nicotinic acetylcholine receptor; NF-κB, nuclear factor kappa-B; MMP9, matrix metalloproteinase nine; p38, ribonuclease P Protein Subunit p38; ERK, extracellular signal-regulated kinase; p70S6K, p70 ribosomal protein S6 kinase; mTOR, mechanistic target of rapamycin; Nrf2, nuclear factor erythroid 2-related Factor 2; HO-1, heme oxygenase-1; α3β4nAChR, α3β4 nicotinic acetylcholine receptor.

## 5 Discussion

Nicotine, a well-known component of cigarettes, is associated with numerous health risks. However, recent research has suggested that nicotine may have beneficial effects, as smokers exhibit lower rates of certain diseases that appear to be associated with nicotine. Here, we provided a comprehensive review of nicotine by discussing its role in various diseases and exploring its toxicity and therapeutic potential.

The effects of nicotine associated with different diseases demonstrate its double-edged characteristics, which may be influenced by diverse factors, including pathophysiological mechanisms, tissue and cell responses, treatment duration, and dosage. Research indicates that the impact of nicotine on animals and humans is primarily mediated via nAChRs. However, it is crucial to recognize that many studies have been limited to the cellular level, potentially differing from the overall effects in the body. Further research, including clinical studies, is necessary to fully understand the therapeutic potential of nicotine in various diseases. In addition, when considering the clinical use of nicotine, safety concerns associated with its complex pharmacological effects, addictive nature, and human tolerance must be considered carefully.

As a known addictive substance, it must be considered that nicotine should be avoided from preventive use despite it shows positive effects in a variety of diseases. Besides, the control of related patches and preparations should be more stringent. In addition, treatment decisions involving patients susceptible to nicotine addiction should be more cautious in clinical applications. Last but most least, there is an urgent need to improve the awareness of the patient population.

There have been numerous reports on the mechanisms of action of nicotine in specific diseases; however, the complexity and diversity of the molecular signals involved make it challenging to cover all regulatory processes clearly and accurately. Notably, many studies have focused on specific molecular signaling pathways associated with nicotine and particular diseases, sometimes leading to conflicting conclusions. Mental diseases such as schizophrenia and MDD are particularly complex due to their unclear pathogenesis, indicating that the pharmacological effects of nicotine in these conditions are still in the exploratory stage. Deeper and more comprehensive research is eagerly anticipated in the future.

Choosing an appropriate method of administration for disease treatment can have a significant impact, ensuring that nicotine does not accumulate in healthy organs. Techniques such as direct injection and atomized administration can achieve a lower dosage and a quicker onset of effects. Inhalation therapy, which involves delivering drugs directly to the airways, has proven effective in treating asthma and COPD and may also benefit individuals with dysphagia and tremors, such as patients with PD. To ensure an effective drug dose in the bronchi and subsequent absorption into the bloodstream, drug preparations with a high proportion of fine particles and consistent and accurate dosing of the active substance are necessary (Sorino et al., 2020).

Moreover, designing precisely targeted drugs for different diseases may be a promising approach to address these issues. Recent studies have explored the synthesis of fully active nanomedicines for targeted cancer treatment (Fang et al., 2023). By incorporating an appropriate targeting group into nicotine, controlled drug release and direct drug enrichment can be achieved, thereby minimizing undesired drug accumulation in nontargeted cells and organs. Further investigation is encouraged to explore this approach and potentially facilitate the clinical use of nicotine in a more controlled and effective manner.

In conclusion, nicotine is widely recognized for its harmful effects; however, recent research has shed light on its potential therapeutic benefits in certain diseases. Consequently, it is eesential to approach nicotine with caution and conduct comprehensive research to fully understand its effects and ensure its safe and effective clinical use. A deeper understanding of the mechanisms of action of nicotine in different diseases and the development of targeted drug delivery systems can pave the way for its use as a medicinal agent.

## Figures and Tables

**Table:** 

**nAChRs**	nicotinic acetylcholine receptors
**SCLC**	small cell lung cancer
**NNK**	4-(methylnitrosamino)-1-(3-pyridyl)-1-butanone
**PKC**	protein kinase C
**MAPK**	mitogen-activated protein kinase
**α-Bgt**	α-bungarotoxin
**ERK**	extracellular signal-regulated kinase
**MEK**	MAPK/ERK kinase
**PI3K**	phosphatidylinositol-3-kinase
**NF-κB**	nuclear factor kappa-B
**VEGF**	vascular endothelial growth factor
**PKB**	protein kinase B
**GSK-3α**	glycogen synthase kinase-3 alpha
**GSK-3β**	glycogen synthase kinase-3 beta
**p70S6K**	p70 ribosomal protein S6 kinase
**4EBP-1**	a binding protein for eukaryotic translation initiation factor 4E
**FKHR**	a member of the forkhead transcription factor family
**DHβE**	dihydro-β-erythroidine
**HB-EGF**	heparin binding-epidermal growth factor
**EGFR**	epidermal growth factor receptor
**cAMP**	cyclic adenosine monophosphate
**TH**	tyrosine hydroxylase
**AP-1**	activator protein-1
**COX-2**	cyclooxygenase-2
**PGE2**	prostaglandin E2
**ECs**	endothelial cells
**SMCs**	smooth muscle cells
**LPS**	lipopolysaccharide
**ACE2**	angiotensin-converting enzyme 2
**VSMC**	vascular smooth muscle cells
**PDGF**	platelet-derived growth factor
**MMPs**	matrix metalloproteinases
**STAT3**	signal transducer and activator of transcription 3
**ROS**	reactive oxygen species
**NLRP3**	NOD-like receptor thermal protein domain associated protein 3
**IL**	interleukin
**ASC**	apoptosis-associated speck-like protein containing CARD
**AMPK**	AMP-activated protein kinase
**COPD**	chronic obstructive pulmonary disease
**PNE**	prenatal nicotine exposure
**TRPCs**	transient receptor potential canonicals
**JNK**	c-Jun N-terminal kinase
**RB**	retinoblastoma protein
**PPARγ**	peroxisome proliferator-activated receptor γ
**AMD**	age-related macular degeneration
**PLD**	phospholipase D
**OA**	osteoarthritis
**HIF-2α**	hypoxia-inducible factor-2α
**JAK2**	Janus kinase 2
**PIN1**	peptidyl-prolyl cis/trans isomerase NIMA-interacting protein 1
**PEDF**	pigment epithelium-derived factor
**AD**	Alzheimer’s disease
**PD**	Parkinson’s disease
**ADHD**	attention-deficit/hyperactivity disorder
**MDD**	major depressive disorder
**TEA**	tetraethyl ammonium
**Aβ**	amyloid beta
**APP**	amyloid precursor protein
**CaM**	calmodulin
**IκBα**	nuclear factor kappa B alpha
**SNP**	single-nucleotide polymorphisms
**NAc**	nucleus accumbens
**rACC**	rostral anterior cingulate cortex
**DS**	dorsal striatum
**DLPFC**	dorsolateral prefrontal cortex
**UC**	ulcerative colitis
**RA**	rheumatoid arthritis
**FLS**	fibroblast-like synoviocytes
**MCP-1**	monocyte chemoattractant protein-1
**TLR**	toll-like receptor
**HMGB1**	high-mobility group box 1
**HO-1**	heme oxygenase-1
**MAdCAM-1**	mucosal vascular addressin cell adhesion molecule-1
**CVB3**	coxsackievirus B3
**IFN-γ**	interferon-γ
**TNF-α**	tumor necrosis factor-alpha
**mTOR**	mechanistic target of rapamycin
**iNOS**	inducible nitric oxide synthase
**E2F**	early two factor.
